# How carbon accounting rules shape incentives for hydrogen production

**DOI:** 10.1038/s41467-026-75473-z

**Published:** 2026-08-10

**Authors:** Gunther Glenk, Philip Holler, Stefan Reichelstein

**Affiliations:** 1 Business School, University of Mannheim, Mannheim, Germany; 2 Center for Energy and Environmental Policy Research, Massachusetts Institute of Technology, Cambridge, MA USA; 3 Graduate School of Business, Stanford University, Stanford, CA USA; 4 ZEW – Leibniz Centre for European Economic Research, Mannheim, Germany

**Keywords:** Business, Economics, Environmental economics

## Abstract

Governments around the world have recently adopted policy support programs for hydrogen, tying the level of support to the assessed carbon intensity of the hydrogen produced. Here we compare alternative carbon accounting rules for determining the policy support available for hydrogen in terms of the resulting financial and carbon emissions performance of Power-to-Gas systems. We calibrate our model to reference plants eligible for the production tax credit available under the Inflation Reduction Act in the United States. Contrary to frequently articulated views, more stringent accounting rules generally provide investors with sufficient incentives to invest in Power-to-Gas systems. Nonetheless, even more stringent rules can lead to carbon intensity levels close to those for hydrogen produced from natural gas with carbon capture. Less stringent rules generally entail stronger investment incentives due to higher profitability, but also significantly higher emissions as investors procure more carbon-intensive electricity from the general grid.

## Abstract (web summary)

Contrary to common views, this paper shows that stringent carbon accounting rules sufficiently incentivize electrolytic hydrogen production yet may yield a carbon intensity near that of hydrogen produced from natural gas with carbon capture.

## Introduction

Governments around the world have recently adopted policies to support electrolytic and other low-carbon hydrogen production technologies^1–5^. These policies aim to accelerate the transition to a decarbonized economy, particularly in hard-to-abate sectors such as steel, chemicals, and heavy-duty transportation^6–11^. Since the abatement potential of electrolytic hydrogen hinges on the emissions embodied in the electricity converted via Power-to-Gas (PtG) processes, governments in the United States (US), the European Union, and other regions have tied the level of policy support to the carbon intensity of the hydrogen produced. Yet, the precise carbon accounting rules for calculating the available policy support remain a topic of intense debate^12–16^.

More stringent accounting rules governing the policy support of electrolytic hydrogen are commonly believed to incentivize hydrogen production during periods of abundant renewable energy and thus result in lower emissions than hydrogen production from natural gas^12,13^. Yet, more stringent rules might also starve PtG systems as long as renewable energy remains infrequent, thereby limiting the profitability of new PtG systems^17,18^. A central question, therefore, is how alternative carbon accounting rules shape the trade-off between the profitability of PtG systems and the life-cycle carbon intensity of the hydrogen produced.

In alignment with Europe, US regulators during the Biden administration announced plans to base the applicable accounting rules on multiple pillars that increase in stringency over time^19^. Accordingly, any renewable electricity investors seek to credit to the produced hydrogen must be deliverable to PtG plants and incremental to the existing renewable energy supply. For hydrogen produced before 2030, the temporal matching of electricity generation and hydrogen production is to be assessed on an annual basis, as is the carbon intensity of hydrogen. After that, the matching requirement is to become more stringent and switch to an hourly basis. Investors may still choose to assess the carbon intensity of hydrogen on either an annual or hourly basis, provided the corresponding annual average does not exceed a certain threshold. On July 4, 2025, the US Congress voted to significantly shorten the timeline for supporting hydrogen production and other clean energy technologies^20^. In particular, the policy support for hydrogen is now limited to investment projects whose construction begins before January 1, 2028. The underlying pillars for assessing the carbon intensity of hydrogen, however, have remained unchanged.

Most recent studies on the policy support for electrolytic hydrogen consider a (central) planner seeking to minimize the total cost of an energy system subject to meeting given demands for electricity and hydrogen^12–16,21,22^. These studies then assess changes in the total cost and carbon emissions of the system depending on whether the hydrogen demand is met by converting incremental renewable energy at select time intervals. A recent review article^15^ points to the value of an analysis wherein a representative investor seeks to maximize the net present value of investments in PtG systems in response to specific policy support mechanisms for electrolytic hydrogen. Such an approach can yield insights into how alternative accounting rules governing the policy support program shape the financial and emissions performance of PtG systems.

This paper examines the impact of alternative accounting rules for assessing the level of policy support available for electrolytic hydrogen. The model is calibrated to reference plants eligible for the production tax credit specified in the Inflation Reduction Act in the context of several US states. Contrary to common expectations, more stringent accounting rules based on hourly electricity matching generally provide investors with sufficient incentives to invest in PtG systems in these states today. Specifically, the analysis estimates internal rates of return between 8.0–15.0% for hydrogen sales prices between $1.0–3.5 per kilogram (kg). These more stringent rules also typically result in life-cycle average carbon intensity levels between about 0.1–9.0 kg of carbon dioxide equivalents per kg of hydrogen (kg CO_2_e per kg H_2_). By comparison, these carbon intensity estimates are lower than those for conventional “gray” hydrogen but, for hydrogen prices above $1.5 per kg, comparable to those for “blue” hydrogen produced from natural gas with carbon capture^23–26^. The wide range of estimates emerging from the analysis reflects that investors will procure increasing amounts of electricity from the general grid as hydrogen prices rise. This effect is particularly pronounced when production tax credits are determined on an hourly basis, as investors then prefer to forgo the tax credit in some hours in favor of converting more general grid power.

Less stringent accounting rules based on annual electricity matching also lead to significantly higher profitability of PtG systems, with most internal rates of return between about 10.0–23.0% for hydrogen prices between $1.0–3.5 per kg. These upper estimates lie substantially above the typical range of investment returns available for renewable energy infrastructure^27–30^. This finding speaks to the frequently voiced concern that tax credits of up to $3.0 per kg could lead to excessive returns for investors^31^. The calculations also project significantly higher life-cycle carbon intensity levels between about 5.0–15.0 kg CO_2_e per kg H_2_. The lower end of this range falls in the middle of estimates for blue hydrogen^23,24^ and emerges for accounting rules that require renewable energy to be incremental. In contrast, the upper end is comparable to estimates for gray hydrogen^26^ and emerges when investors can also credit renewable energy from existing sources. Relative to more stringent accounting rules, the higher estimates for both profitability and carbon intensity now reflect investors’ response to convert substantially more carbon-intensive electricity from the general grid.

This paper contributes to the emerging literature on the role of carbon accounting in determining the effectiveness of climate policies^32–35^. The analysis also relates to recent work on the role of product carbon footprints in decarbonizing supply chains^36^. Reliable product carbon footprints are increasingly demanded by corporate customers seeking to decarbonize their supplier network^37^. Similarly, the Carbon Border Adjustment Mechanism by the European Union, set to take effect in 2026, requires an assessment of the emissions embodied in certain goods imported to the bloc^38^. In this context, the present analysis provides insights into how alternative rules for calculating the emissions embodied in products affect the investment decisions of producers outside the European Union.

## Results

### Economic model

**Fig. 1:**
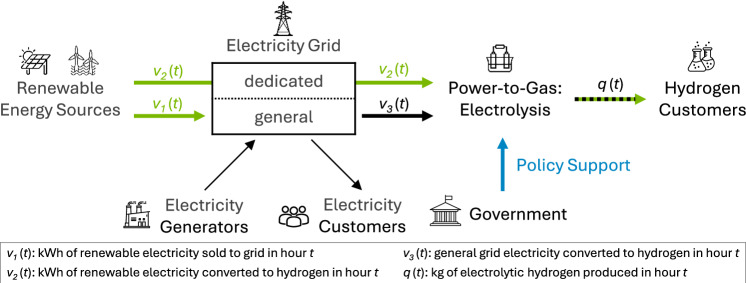
Techno-economic setting. This figure illustrates the techno-economic setting of the PtG system considered in the analysis.

Consider an investor seeking to maximize the net present value of an investment in a PtG system converting electricity to hydrogen (Fig. 1). This investment may include renewable energy sources (wind, solar, or both) for the electricity converted to hydrogen. The analysis would be essentially unchanged if the investor were to procure renewable electricity from a third party, possibly via a power purchasing agreement. In that case, the renewables and the PtG plant (electrolyzer, compressor, and piping system) could be located in different places, yet the renewable energy generated can be delivered to the PtG plant, provided the parties are connected to the same grid.

To maximize the value obtained from such a PtG system, the investor seeks to size the capacity of the renewables optimally in relation to the size of the PtG plant and thereafter use the installed capacity optimally (see “Methods” for details). At any given point in time, the generated renewable electricity can be sold into the grid at the current market price, or it can be transferred to the PtG plant for hydrogen production, even in the absence of a direct transmission line. Such transfers can be certified via Energy Attribute Certificates (EACs) for renewable energy, as envisioned in recent regulatory guidance^19^. The PtG plant can procure additional electricity from the general pool to utilize any spare capacity. Yet, to the extent that such grid electricity was generated from fossil fuels, it will be burdened with carbon emissions. Each kg of hydrogen produced can be sold to customers at a fixed price and may qualify for a particular level of policy support, depending on the underlying accounting rules.

This analysis examines the impact that alternative accounting rules have on determining the level of policy support available to PtG systems. The applicable rules determine how much grid electricity the PtG plant can utilize to keep the emissions attributed to the hydrogen produced below a certain threshold in order to qualify the hydrogen produced for a certain level of policy support. The hydrogen produced and the corresponding level of policy support, in turn, determine the cash flows associated with the project.

To examine the impact of alternative accounting rules, this analysis assesses the profitability of PtG systems and thus the financial incentives for investors to deploy them. Profitability is measured as the cost of capital at which the maximized net present value of the PtG system would be equal to zero. The cost of capital, therefore, reflects the internal rate of return of the investment and allows us to compare PtG systems of different sizes.

As a second performance criterion, the average carbon intensity of the hydrogen produced over the entire life cycle of a PtG system is also assessed. This carbon intensity is measured as the total emissions embodied in the electricity from the general pool converted to hydrogen over the entire life cycle of the system, divided by the total hydrogen produced. Excluding any emissions embodied in the installed equipment, the metric captures the well-to-gate Scope 2 emissions of electrolytic hydrogen.

To formalize this life-cycle carbon intensity measure of electrolytic hydrogen, suppose that policy support for hydrogen produced is determined according to some generic accounting rule *R*. This analysis considers four distinct accounting rules, denoted by the letters *A* through *D*, respectively. Assuming the generic accounting rule *R* applies, let *v*_1*i*_(*t*∣*R*) denote the optimized kilowatt-hours (kWh) of renewable electricity sold to the general grid in hour *t* of year *i*. Here *t* = 1, …, *m* for *m* = 8760 hours, and *i* = 1, …, *T* for *T* useful years of the investment. Let *v*_2*i*_(*t*∣*R*) further denote the optimized kWh of dedicated renewable electricity converted to hydrogen and *v*_3*i*_(*t*∣*R*) the optimized kWh of general grid electricity converted to hydrogen in hour *t* of year *i*, for the given accounting rule *R*. We further denote by *CI*_*ei*_(*t*) the capacity-weighted average carbon intensity of general grid electricity in hour *t* of year *i* (in kg CO_2_e per kWh) and by *η* the average conversion efficiency of the PtG plant (in kg H_2_ per kWh). Given the underlying accounting rule *R*, the life cycle carbon intensity (in kg CO_2_e per kg H_2_) of the hydrogen produced then is:

1 $$C{I}_{h}(R)\equiv \frac{{\sum }_{i=1}^{T}{\sum }_{t=1}^{m}C{I}_{ei}(t)\cdot {v}_{3i}(t| R)}{\mathop{\sum }_{i=1}^{T}\mathop{\sum }_{t=1}^{m}{q}_{i}(t| R)},$$

where *q*_*i*_(*t*∣*R*) = *η* ⋅ [*v*_2*i*_(*t*∣*R*) + *v*_3*i*_(*t*∣*R*)] denotes the optimized kg of hydrogen produced in hour *t* of year *i*.

Consistent with recent regulatory guidance^19^, the life-cycle carbon intensity measure in equation (1) initially adopts a market-based approach that assesses the emissions embodied in electrolytic hydrogen based on contractual arrangements for renewable energy supply. Yet, the analysis also examines the impact of a location-based approach, where any electricity procured from the grid is assigned the average carbon intensity of the local grid; see the Discussion. Marginal rather than average emission factors are also examined there.

The model is calibrated to reference plants eligible for the production tax credit as specified in the Inflation Reduction Act of the US. The Inflation Reduction Act^1^ provides a tax credit of up to $3.0 per kg for hydrogen with a carbon intensity of up to 0.45 kg CO_2_e per kg H_2_. The analysis is initially conducted for the state of Texas, as this state has seen significant growth in wind and solar energy capacity in recent years. It is also home to several industries that require hydrogen as a production input^39^ and to large-scale hydrogen production projects supported by the Inflation Reduction Act^40^. The Discussion assesses the sensitivity of the findings to changes in input parameters, including lower production tax credits, changes in future electricity prices and grid carbon intensity levels, changes in the degradation of capacity, and lower system prices of PtG units. We further examine the robustness of the findings by considering US states other than Texas. The data inputs come from multiple sources, including journal articles, technical reports, and industry databases (see “Methods” for details).

### Carbon intensity of electricity based on hourly matching

Since most renewable energy generation is intermittent, a key point of policy debate is the time interval for matching electricity generation and hydrogen production. Analysts have argued that hourly matching incentivizes electrolytic hydrogen production during periods of abundant renewable energy and thus at lower emissions than hydrogen production from natural gas^12,13^. Investors have contended that this might starve PtG systems if only intermittent renewable energy sources are available, thereby limiting the profitability of the system^17,18^.

In recent guidance, US regulators have announced plans to require hourly electricity matching for hydrogen produced from 2030 onward^19^. Yet until 2030, investors can choose to assess the carbon intensity of hydrogen on either an annual basis or an hourly basis, provided the corresponding annual average does not exceed 4.0 kg CO_2_e per kg H_2_. This flexibility, regulators have argued, provides investors with “additional investment certainty” if they cannot procure renewable energy for a limited number of hours during the year.

This subsection examines two accounting rules that are both consistent with the recent guidance. The first rule, denoted by *A*, calculates tax credits on an hourly basis. Specifically, the carbon intensity of hydrogen in hour *t* of year *i* is given by equation (2):

2 $$C{I}_{hi}(t| A)\equiv \frac{C{I}_{ei}(t)\cdot {v}_{3i}(t| A)}{{q}_{i}(t| A)}.$$

Further, the annual tax credit for the PtG system in year *i* is given by multiplying the hourly amount of hydrogen produced by the production tax credit corresponding to the assessed carbon intensity of hydrogen for that hour and summing over all hours of the year, as given by:

3 $$PT{C}_{hi}(A)\equiv \mathop{\sum }_{t=1}^{m}f\left(C{I}_{hi}(t| A)\right)\cdot {q}_{i}(t| A),$$

where *f*( ⋅ ) identifies the tax credit (in $ per kg H_2_) corresponding to a given carbon intensity of hydrogen (in kg CO_2_e per kg H_2_), as specified in the Inflation Reduction Act^1^. If PtG systems satisfy the prevailing wage and apprenticeship requirements, *f*( ⋅ ) is given by:

4 $$f(x)\equiv \left\{\begin{array}{ll}3.0\quad &\,{{{\rm{if}}}}\,x\le 0.45,\\ 1.0\quad &\,{{{\rm{if}}}}\,x\in (0.45,1.5],\\ 0.75\quad &\,{{{\rm{if}}}}\,x\in (1.5,2.5],\\ 0.6\quad &\,{{{\rm{if}}}}\,x\in (2.5,4.0],\\ 0.0\quad &\,{{{\rm{if}}}}\,x > 4.0.\end{array}\right.$$

As an alternative to rule A, tax credits can be calculated on the basis of the annual carbon intensity of hydrogen produced. Referring to this approach as rule *B*, the annual carbon intensity of hydrogen is given by equation (5):

5 $$C{I}_{hi}(B)\equiv \frac{{\sum }_{t=1}^{m}C{I}_{ei}(t)\cdot {v}_{3i}(t| B)}{\mathop{\sum }_{t=1}^{m}{q}_{i}(t| B)},$$

and

6 $$PT{C}_{hi}(B)\equiv f\left(C{I}_{hi}(B)\right)\cdot \mathop{\sum }_{t=1}^{m}{q}_{i}(t| B).$$

A direct comparison of equations (3) and (6) shows the operational flexibility gain under accounting rule *A* in comparison to *B*. Rule A allows investors to forgo tax credits in some hours and fully utilize the PtG plant with general grid electricity without compromising tax credit eligibility in other hours.

**Fig. 2:**
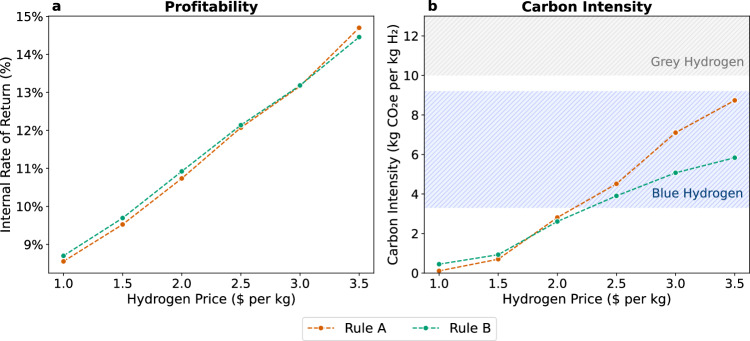
Life-cycle performance resulting from hourly electricity matching. This figure shows the impact of accounting rules *A* (hourly tax credits) and *B* (annual tax credits) on (**a**) the profitability of PtG systems, and (**b**) the life-cycle average carbon intensity of hydrogen, given hydrogen prices between $1.0 and $3.5 per kg. The life-cycle carbon intensity metric is calculated in accordance with equation (1). Dots show our point estimates at specific hydrogen prices, while dashed lines interpolate between them for illustration.

**Fig. 3:**
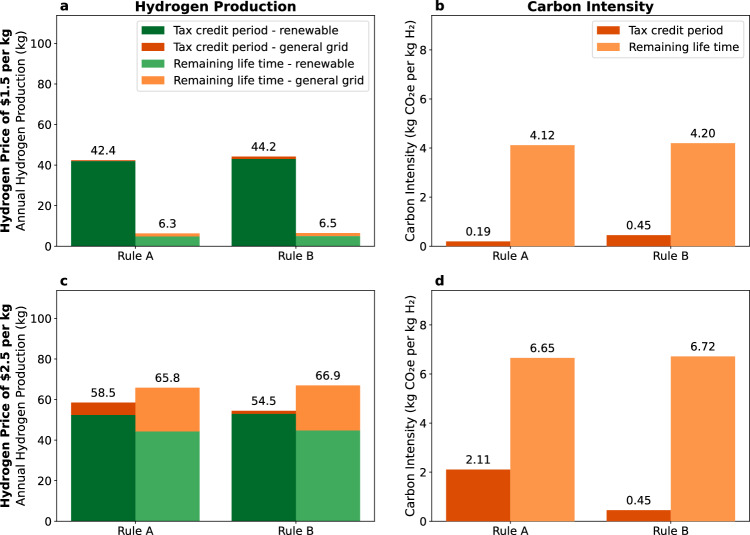
Stage performance resulting from hourly electricity matching. This figure shows the impact of accounting rules *A* (hourly tax credits) and *B* (annual tax credits) on (**a**, **c**) the annual hydrogen production and (**b**, **d**) the annual carbon intensity of hydrogen, given hydrogen prices of $1.5 and $2.5 per kg. Annual hydrogen production is calculated based on a renewable power generation capacity of 1.0 kilowatt peak (see “Methods” for details).

Figures 2, 3 display our estimates for the impact of the two accounting rules on profitability and the life-cycle carbon intensity of hydrogen. Specifically, Fig. 2 shows the performance of PtG systems across hydrogen prices ranging from $1.0 to $3.5 per kg. For ease of computational burden, the results are displayed in incremental steps of $0.5 per kg. These prices reflect the range of transaction prices observed for industrial-scale hydrogen supply today and are often cited as critical benchmarks for the widespread adoption of low-carbon hydrogen^41,42^. Figure 3 shows our estimates at hydrogen prices of $1.5 and $2.5 per kg, split between the two principal stages of PtG systems: the first ten years, when they are eligible for the tax credit, and their remaining lifetime.

Contrary to commonly voiced concerns, both accounting rules will make electrolyzer investments in Texas profitable today (Fig. 2a). In particular, the calculations project that the internal rate of return of PtG systems increases almost linearly from 8.6% to 14.7% as hydrogen prices rise from $1.0 to $3.5 per kg and the PtG systems produce more hydrogen, especially after the tax credit (Fig. 3a, c). These returns are close to the typical range of 8.5–12.5% that have been observed for early investments in renewable energy infrastructure^27–30^.

The profitability of investments is also similar under both accounting rules. In other words, the operational flexibility under accounting rule *A* (hourly tax credits) does not translate into higher profitability. The nearly identical profitability levels emerge because, at lower hydrogen prices, PtG systems operate in similar ways under both rules (Fig. 3a, c). Given rule *A*, rising hydrogen prices imply that PtG systems will forgo the tax credit in more hours and produce more hydrogen from general grid electricity. Yet, this financial benefit in some hours is effectively offset by PtG systems that earn the tax credit for all hydrogen produced in a given year according to rule *B*.

As for emissions, both accounting rules A and B result in a lower life-cycle carbon intensity than conventional hydrogen production, but not necessarily much lower (Fig. 2b). For hydrogen prices up to $1.5 per kg, the calculations project a carbon intensity between 0.1–0.9 kg CO_2_e per kg H_2_ for PtG systems located in Texas under both accounting rules. For prices above $1.5 per kg, the carbon intensity ranges between 2.8–8.7 kg CO_2_e per kg H_2_ under rule *A* and 2.6–5.8 kg CO_2_e per kg H_2_ under rule *B*. These estimates are lower than those for conventional (gray) hydrogen but comparable to those for (blue) hydrogen produced from natural gas with carbon capture^23,24,26^. The increase in the life-cycle average carbon intensity arises because PtG systems produce more hydrogen from general grid electricity once the tax credit eligibility expires (Fig. 3a, c), which increases the carbon intensity of hydrogen during that period (Fig. 3b, d). PtG systems also boost hydrogen output when prices rise, especially after the tax credit (Fig. 3a, c), which increases the weight of that period in the life-cycle average. Such projections must, of course, be qualified by their reference to the current carbon intensity of general grid electricity (see the Discussion).

The calculations also show that, for hydrogen prices above $1.5 per kg, the carbon intensity of hydrogen increases more sharply with hourly tax credits (rule *A*) than with annual tax credits (rule *B*). This reflects that PtG systems under rule *A* are incentivized to forgo the tax credit more often in favor of a higher conversion of general grid power (see Supplementary Note 2 for details). Nevertheless, the annual carbon intensity of hydrogen remains well below 4.0 kg CO_2_e per kg H_2_ for the hydrogen prices considered (Fig. 3b, d). The constraint on the hourly assessment of the carbon intensity of hydrogen thus remains non-binding. Under rule *B*, the carbon intensity of hydrogen during the tax credit period is always exactly equal to 0.45 kg CO_2_e per kg H_2_, the threshold for the highest tax credit (see equation (4)).

### Carbon intensity of electricity based on annual matching

In response to concerns about hourly matching, US regulators^19^ have indicated their willingness to assess both the temporal matching of electricity and the carbon intensity of hydrogen on an annual basis for hydrogen produced through 2030. This accounting rule, denoted by *C*, effectively allows investors to offset renewable energy sold to the general grid in some hours against electricity procured from that grid in other hours. With $C{I}_{ei}=\frac{1}{m}{\sum }_{t=1}^{m}C{I}_{ei}(t)$ representing the annual average carbon intensity of grid electricity, the average carbon intensity of hydrogen produced in year *i* is given by equation (7):

7 $$C{I}_{hi}(C)\equiv \frac{C{I}_{ei}\cdot {\sum }_{t=1}^{m}\left({v}_{3i}(t| C)-{v}_{1i}(t| C)\right)}{\mathop{\sum }_{t=1}^{m}{q}_{i}(t| C)}.$$

Tax credits are again calculated on an annual basis, as described in equation (6). The analysis initially assumes that any such rule applies for the entire life of a PtG system.

Following recent regulatory guidance^19^, the analysis has so far required investors to install incremental renewable energy capacity. Since this requirement remains controversial^13^, a further accounting rule, denoted by *D*, does not mandate co-investment in renewables. Instead, investors have a choice on whether to co-invest in renewables or to procure EACs for renewable energy (also often referred to as Renewable Energy Certificates) on the open market to offset electricity procured from the general grid. Since most of the EACs are not matched with specific time intervals, the resulting carbon intensity of hydrogen produced in year *i* is given by equation (8):

8 $$C{I}_{hi}(D)\equiv \frac{C{I}_{ei}\cdot \left[\mathop{\sum }_{t=1}^{m}\left({v}_{3i}(t| D)-{v}_{1i}(t| D)\right)-{v}_{ri}\right]}{\mathop{\sum }_{t=1}^{m}{q}_{i}(t| D)},$$

where *v*_*ri*_ denotes the optimized amount of EACs (in kWh) procured in year *i*. The annual tax credit for the PtG system is again calculated as described in equation (6).

**Fig. 4:**
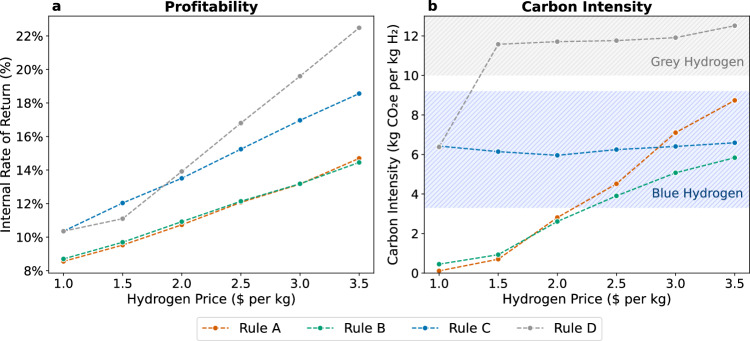
Life-cycle performance resulting from annual electricity matching. This figure shows the impact of accounting rules *C* (incremental renewable energy) and *D* (non-incremental renewable energy) on (**a**) the profitability of PtG systems, and (**b**) the life-cycle average carbon intensity of hydrogen, given hydrogen prices between $1.0 and $3.5 per kg. The life-cycle carbon intensity metric is calculated in accordance with equation (1). Dots show our point estimates at specific hydrogen prices, while dashed lines interpolate between them for illustration. Results for accounting rules *A* and *B* are shown for reference.

**Fig. 5:**
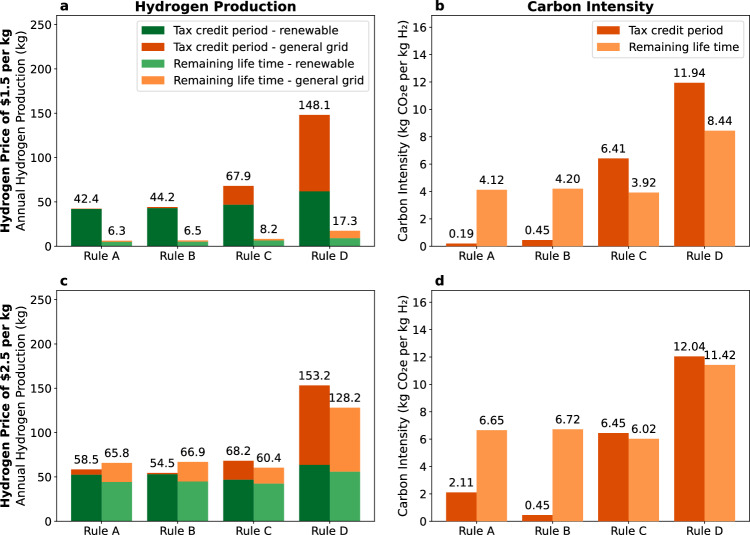
Stage performance resulting from annual electricity matching. This figure shows the impact of accounting rules *C* (incremental renewable energy) and *D* (non-incremental renewable energy) on (**a**, **c**) the annual hydrogen production and (**b**, **d**) the annual carbon intensity of hydrogen, given hydrogen prices of $1.5 and $2.5 per kg. Annual hydrogen production is calculated based on a renewable power generation capacity of 1.0 kilowatt peak (see “Methods” for details). Results for accounting rules *A* and *B* are shown for reference.

Figures 4, 5 illustrate the impact of accounting rules *C* and *D*. Both rules lead to substantially higher profitability of PtG systems than the two accounting rules based on hourly electricity matching (Fig. 4a). In particular, the calculations project that the internal rate of return of PtG systems located in Texas increases from 10.4% under both rules to 18.6% under rule *C* and 22.5% under rule *D* as hydrogen prices rise from $1.0 to $3.5 per kg (Fig. 5a, c). These upper estimates lie substantially above the typical range of 8.5–12.5% observable for early investments in renewable energy infrastructure^27–30^. This speaks to the frequently voiced concern that tax credits of up to $3.0 per kg might be excessive, leading to abnormal investment returns^31^.

The analysis also shows that, as hydrogen prices rise, the profitability of PtG systems grows faster if non-incremental renewable energy is permitted (rule *D*). Investors can then exploit the effective price arbitrage opportunities between EACs and tax credits, resulting in larger PtG plants and higher hydrogen production (Fig. 5a, c). For hydrogen prices up to $2.0 per kg, higher revenues and capital costs under rule *D* roughly offset each other, resulting in similar internal rates of return as under rule *C*. There will always be co-investment in renewables under rule *D*, reflecting that such investments are profitable on their own, irrespective of any production tax credits.

Regarding emissions, accounting rule *C* results in substantially lower life-cycle carbon intensity levels than rule *D*, provided hydrogen prices are above $1.5 per kg (Fig. 4b). Under rule *C*, the calculations point to a carbon intensity of about 6 kg CO_2_e per kg H_2_ for PtG systems located in Texas. This value falls right in the middle of estimates for blue hydrogen^23,24^. Under rule *D*, the carbon intensity is about 12 kg CO_2_e per kg H_2_ for hydrogen prices above $1.0 per kg, which is comparable to lower estimates for gray hydrogen^26^. The higher value under rule *D* reflects investors’ tendency to build larger PtG systems, produce more hydrogen, and convert significantly more grid electricity both during the tax credit period and thereafter (Fig. 5). Note that, at a hydrogen price of $1.0 per kg, the calculations also yield a carbon intensity of about 6 kg CO_2_e per kg H_2_ under rule *D* because, at that price, it is economically unattractive for investors to procure any EACs for renewable energy on the open market.

The calculations project that the life-cycle carbon intensity levels under both rules *C* and *D* remain fairly stable across hydrogen prices above $1.0 per kg. Under rule *C*, this stability mainly reflects the natural limit imposed by the amount of self-generated renewable energy (Fig. 5a, c). Under rule *D*, the PtG plant will procure a corresponding share of electricity from the general grid (see Supplementary Note 2 for details). Finally, under rule *C*, the carbon intensity of hydrogen is generally at least as large as that emerging from rule *B*, with the difference reflecting the impact of annual versus hourly matching.

## Discussion

Governments often evaluate policy support programs in terms of their impact relative to their costs. In this context, the policy impact of the tax credit is measured as the total hydrogen produced over the life cycle of a PtG system divided by the discounted value of the annual tax credits awarded by the government (equation (15) in Methods).

**Fig. 6:**
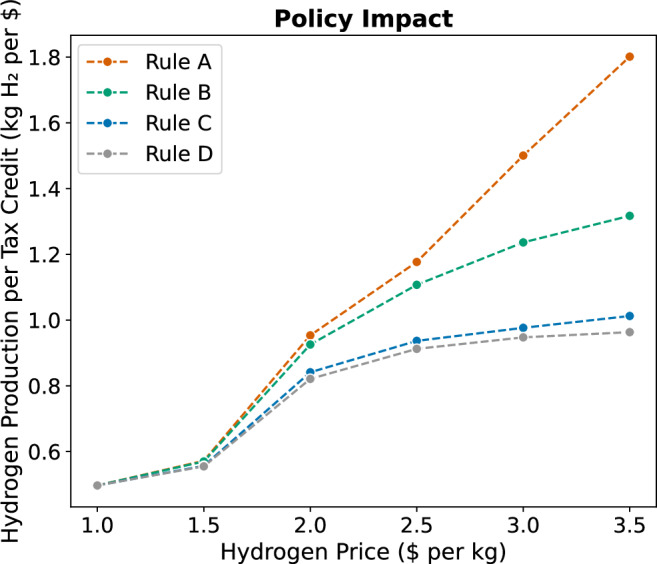
Policy impact of alternative accounting rules. This figure shows the impact of alternative carbon accounting rules on the capacity of the policy support to incentivize electrolytic hydrogen production, given hydrogen prices between $1.0 and $3.5 per kg. Dots show our point estimates at specific hydrogen prices, while dashed lines interpolate between them for illustration.

Figure 6 shows that, for the entire range of hydrogen prices considered here, a tax credit of $3.0 per kg induces the production of more than 1.0 kg of hydrogen. This conclusion emerges for each of the four carbon accounting rules considered above. For hydrogen prices up to $1.5 per kg, the policy impact of all four rules is moreover nearly identical. For prices above $1.5 per kg, the policy impact increases at a decreasing rate under rules *B* and *D*. This pattern reflects the financial incentive to convert more electricity to hydrogen as hydrogen prices rise, yet at a decreasing rate as PtG plants reach their capacity limit more often. The particularly sharp increase observed for rule *A* occurs mainly because PtG systems will then forgo the tax credit more often and convert more general grid electricity. Nevertheless, PtG systems produce nearly the same amount of hydrogen over their life cycle as under rule *B* (Fig. 5a, c). The lower increase under accounting rules *C* and *D* mainly occurs because the total tax credit received remains fairly stable across hydrogen prices, and the life-cycle amount of hydrogen produced increases less strongly in comparison to rules *A* and *B*.

Recent regulatory guidance^19^ calls for investors to obtain and retire EACs in order to verify that a kWh of energy has been generated from renewables. Most EACs available today are traded separately from the underlying electricity^32^. Thus, renewable electricity can be sold to one customer and the corresponding EACs to another. Analysts have argued that such unbundled trading undermines incentives for renewable energy deployment and that EACs should be tied (or bundled) to the electricity they certify^32^. While as of today there is no regulatory guidance on this issue, accounting rules *A* and *B* effectively reflect *bundled* EACs, whereas rules *C* and *D* reflect *unbundled* EACs.

Consistent with recent regulatory guidance^19^, the approach has allowed investors to assess the emissions embodied in the electricity they consume based on contractual arrangements with energy suppliers. Critics often argue, however, that such *market-based* methods can also enable firms to misrepresent the actual emissions embodied in their electricity consumption^32^. Some further advocate for using *location-based* methods instead, where any electricity procured from the grid is assigned the average carbon intensity of the local grid^43^. Such methods effectively compel investors to co-locate renewables with the PtG plant and to directly connect them with dedicated transmission lines. Without such co-location, any renewable electricity produced would have to flow through the local grid and would incur grid fees. In addition, the PtG plant would only be credited with a fraction of this renewable supply, while investors would bear the full cost of developing the renewable capacity.

To examine the impact of location-based methods in more detail, we assume that investors can co-locate renewables with the PtG plant and obtain the same wind and solar resources as before. Clearly, this is a strong assumption insofar as areas near hydrogen customers may have less space or weaker wind and solar irradiation. The analysis in Supplementary Note 4 focuses on accounting rules *A*, *B*, and *C*, since rule *D* reflects market-based methods by construction. We find that the performance of PtG systems under each of these three rules essentially parallels the findings shown in Fig. 4. In particular, the profitability of PtG systems is slightly higher, the life-cycle average carbon intensity of hydrogen is roughly equivalent, and the policy impact is slightly higher. Any differences are primarily due to the avoidance of grid charges on generated renewable energy under location-based methods. As a result, investors build relatively more renewable energy capacity, a larger PtG component, and convert relatively more renewable energy, especially after the tax credit period. Note also that if PtG plants could not be co-located with renewables, location-based approaches would result in no investments provided the carbon intensity of grid electricity were to stay at present levels.

Supplementary Note 5 examines the sensitivity of the findings to changes in policy design, market conditions, and technology inputs. While the absolute levels of profitability and emissions will naturally vary, the relative impact of the four carbon accounting rules remains robust across the variations considered. As one would expect, lower production tax credits reduce the profitability of PtG systems but increase the policy impact of the tax credits across all carbon accounting rules. In contrast, the life-cycle carbon intensities of hydrogen are fairly insensitive to changes in production tax credits.

Turning to future electricity market conditions, the analysis examines the impact of grid electricity with lower carbon intensity. Specifically, we allow for lower averages and a smaller variance of electricity market prices. The calculations in Supplementary Notes 6–8 show that the profitability of PtG systems and the policy impact of the tax credits remain unchanged by lower future carbon intensity levels of general grid electricity, while the life-cycle carbon intensity of hydrogen declines notably for all four of the accounting rules considered above. The profitability of PtG systems is also relatively insensitive to changes in future electricity prices, specifically at higher hydrogen prices. Higher (lower) electricity prices result in more (less) renewable sales and less (more) grid conversion, partially hedging price movements. Yet, the life-cycle carbon intensity of hydrogen and the policy impact of the tax credits tend to decline (strongly increase) with increasing (decreasing) future electricity prices.

The analysis is also repeated in the context of California, Iowa, and New York. These states differ substantially in their power generation mixes, electricity wholesale market prices, carbon intensity levels of general grid electricity, and the availability of wind and solar resources. The calculations in Supplementary Note 9 show that for each of the four applicable accounting rules, the profitability of PtG systems in California and Iowa is fairly similar, but notably lower than in Texas. In New York, the profitability of PtG systems is generally even lower. The life-cycle carbon intensities of hydrogen are relatively similar across Texas, California, and New York under all accounting rules. In Iowa, however, carbon intensity estimates are slightly lower under accounting rules *A*–*C* and notably higher under rule *D* due to the higher carbon intensity of general grid electricity. The policy impact of the tax credit is also fairly similar across all states under the four accounting rules considered.

Varying the degradation rates of the electrolyzer in Supplementary Note 10 shows that the profitability of PtG systems is slightly higher (lower) when the annual degradation rate is lower (higher) than in the reference scenario, reflecting that usable capacity becomes slightly more (less) productive. The life-cycle carbon intensity of hydrogen and the policy impact of the tax credit remain almost unchanged.

The analysis is repeated for alkaline electrolyzers rather than polymer electrolyte membrane (PEM) electrolyzers. As detailed in Supplementary Note 11, the profitability of PtG systems, the life-cycle carbon intensity of hydrogen, and the policy impact of the tax credits are generally higher for alkaline electrolyzers. These increases mainly reflect that alkaline electrolyzers currently have lower system prices than PEM electrolyzers. This price differential results in larger electrolyzers and the conversion of more grid electricity. Regarding alternative electrolyzer costs, Supplementary Note 12 shows the rate of change at which the profitability, the life-cycle carbon intensity of hydrogen produced, and the policy impact of the production tax credits increase with lower electrolyzer system prices.

Finally, the analysis explores the impact of marginal emission factors for general grid electricity. Supplementary Note 13 shows that the profitability of PtG systems and the policy impact of the tax credit based on either marginal or average emission rates are almost identical. In contrast, the life-cycle average carbon intensity of hydrogen is significantly higher when marginal rather than average emission rates are used. This finding reflects that, by construction, marginal emission rates are at least as high as average emission rates. Overall, these sensitivity tests suggest that variations in the basic input parameters alter the absolute life-cycle performance of PtG systems but not the relative impact of alternative carbon accounting rules.

In addition to the preceding sensitivity tests, the framework lends itself to several immediate extensions. For example, investors could have the option of installing an energy storage unit. Provided that such an addition is financially attractive, it would likely improve the life-cycle carbon intensity of hydrogen produced and the policy impact of the tax credit. This would mainly reflect that more of the generated renewable energy could be converted to hydrogen or sold to the grid at higher prices. Alternatively, disconnecting the PtG system from the grid would result in hydrogen with a carbon intensity of zero. Profitability, however, would be reduced because any excess renewable energy could not be sold to the grid, while the PtG unit would be idle at some hours of the year. Finally, electrolyzers might be constrained in their allowable load range. Such a constraint would result in less hydrogen being produced, thereby reducing both the profitability of PtG systems and the policy impact of the tax credit.

Future research on the policy support for low-carbon energy technologies could explore the implications of other carbon accounting rules. The analysis has focused on those widely discussed and described in regulatory guidance, but other approaches are readily conceivable as well. Future work could also adapt the model to other jurisdictions that support electrolytic hydrogen. In the European Union, for example, support levels depend not only on the carbon intensity of hydrogen but also on the outcomes of competitive auctions^2^. Finally, it would be instructive to integrate our framework with prior studies^12–16,21^ that have developed (partial) market equilibrium models with given price elasticities of electricity and hydrogen demand. Such extended models should provide additional insights into the economic implications of alternative accounting rules for assessing the carbon intensity of hydrogen.

## Methods

### Economic model

The model considers an investor seeking to maximize the net present value of an investment in a PtG system. The upfront investment problem entails the capacity configuration in terms of the joint capacity size of the renewables, the share of this capacity constituted by wind energy, and the capacity size of the PtG plant. Given the capacity configuration, the inner optimization seeks to maximize the annual contribution margin through an optimized real-time use of the installed capacity. The decision variables include the kWh of renewable energy sold to the general grid, the kWh of renewable energy contractually dedicated to the PtG plant, and the kWh of electricity procured from the general grid. The subsequent derivations initially focus on accounting rule *A*.

To describe the inner optimization, let ${v}_{1i}^{\circ }(t| A)$ denote the kWh of renewable electricity sold to the general grid and ${v}_{2i}^{\circ }(t| A)$ the kWh of dedicated renewable electricity converted to hydrogen in hour *t* of year *i*, given rule *A*. We also denote by *k*_*e*_ ∈ [0, 1] the peak capacity of the renewables mix and by *s* ∈ [0, 1] the share of this peak capacity constituted by wind energy. For the purpose of the economic model, we normalize the capacity investment in renewables to 1 kilowatt (kW) of joint peak electricity generation. To be sure, the numerical analysis calibrates the attainable costs and revenues of renewables and PtG plants in accordance with system sizes that have actually been built in recent years. We further denote by *CF*_*wi*_(*t*), *CF*_*si*_(*t*) ∈ [0, 1], respectively, the capacity factors, that is, the shares of the maximum wind and solar power generation in hour *t* of year *i*. The actual amount of renewable power generated in hour *t* of year *i* is then given by *CF*_*i*_(*t*∣*s*) ⋅ *k*_*e*_ ⋅ 1hour, where *CF*_*i*_(*t*∣*s*) = *s* ⋅ *CF*_*wi*_(*t*) + (1 − *s*) ⋅ *CF*_*si*_(*t*).

Let *p*_*si*_(*t*) denote the price per kWh at which renewable energy can be sold on the open market in hour *t* of year *i*. Wind and solar power in the US are both eligible for production tax credits in the after-tax amount of *PTC*_*ei*_ per kWh of power generated in year *i*. Since the production tax credits are only available for the first ten years of the investment, *PTC*_*ei*_ = 0 for the remaining lifetime. To account for the impact of corporate income taxes, we denote the investor’s effective income tax rate by *α* ∈ (0, 1). The pre-tax contribution margin from renewable power generation of the PtG system in year *i* can be expressed as:

9 $$C{M}_{ei}(A)\equiv \mathop{\sum }_{t=1}^{m}\left({p}_{si}(t)+\frac{PT{C}_{ei}}{1-\alpha }\right)\cdot \left({v}_{1i}^{\circ }(t| A)+{v}_{2i}^{\circ }(t| A)\right),$$

where the amounts of renewable energy sold to the general grid and converted to hydrogen can be chosen such that they jointly do not exceed the amount generated at any given time. Formally,

10 $${v}_{1i}^{\circ }(t| A)+{v}_{2i}^{\circ }(t| A)\le C{F}_{i}(t| s)\cdot {k}_{e}\cdot 1\,{{{\rm{hour}}}}\,,\,\,{{{\rm{for}}}}\, {{{\rm{all}}}}\,\,{{t}}=1,\ldots,{{m}}.$$

In addition to converting renewable energy, the electrolyzer can procure grid electricity up to its capacity limit. Let ${v}_{3i}^{\circ }(t| A)$ denote the kWh of general grid electricity converted to hydrogen in hour *t* of year *i*, given rule *A*. Let *p*_*bi*_(*t*) further denote the price per kWh at which general grid electricity can be bought on the market in hour *t* of year *i*. To produce hydrogen, the PtG plant also incurs a variable cost of *δ*_*e*_ for every kWh of renewable energy transmitted to the PtG plant via the grid. This cost markup includes grid surcharges and other retail charges for large-scale industrial customers. The PtG plant further incurs a variable cost of *w*_*h*_ per kg of hydrogen produced for consumable inputs, such as water and reactants for deionizing the water.

Every kg of hydrogen produced can be sold to customers at a fixed price of *p*_*h*_ and may qualify for a production tax credit, the magnitude of which depends on its assessed carbon intensity. We denote by $PT{C}_{hi}^{\circ }(A)$ the after-tax amount of the annual tax credit for the PtG system in year *i* and calculate it in direct analogy to equation (3). Since this tax credit is available only for the first ten years of the investment, *PTC*_*hi*_(*A*) = 0 for the remaining lifetime. With *k*_*h*_ denoting the kW of peak power absorption of the PtG plant, the pre-tax contribution margin from hydrogen production in year *i* can be expressed as:

11 $$C{M}_{hi}(A) \equiv 	 \frac{PT{C}_{hi}^{\circ }(A)}{1-\alpha }+\mathop{\sum}_{t=1}^{m}\left({p}_{h}-{w}_{h}\right)\cdot \eta \cdot \left({v}_{2i}^{\circ }(t| A)+{v}_{3i}^{\circ }(t| A)\right) \\ 	 - \left({p}_{si}(t)+{\delta }_{e}\right)\cdot {v}_{2i}^{\circ }(t| A)-{p}_{bi}(t)\cdot {v}_{3i}^{\circ }(t| A),$$

where the amounts of dedicated renewable and general grid electricity can be chosen such that they do not exceed the peak capacity of the PtG plant at any given time. Formally,

12 $${v}_{2i}^{\circ }(t| A)+{v}_{3i}^{\circ }(t| A)\le {k}_{h}\cdot 1\,{{{\rm{hour}}}}\,,\,\,{{{\rm{for}}}}\, {{{\rm{all}}}}\,\,{{t}}=1,\ldots,m.$$

Note in passing that both ${v}_{2i}^{\circ }(t| A)$ and ${v}_{3i}^{\circ }(t| A)$ can be chosen flexibly for each hour of the year, since the PtG technology considered in the numerical analysis can be ramped up and down rapidly^44^.

Aggregating the components in equations (9) and (11) gives the total annual pre-tax contribution margin of the PtG system in year *i*. The inner optimization problem can then be expressed as:

13 $$C{M}_{i}(s,{k}_{e},{k}_{h}| A)\equiv {{\max }_{{v}_{1i}^{\circ }(t| A),\,{v}_{2i}^{\circ }(t| A),\,{v}_{3i}^{\circ }(t| A)}}\left\{C{M}_{ei}(A)+C{M}_{hi}(A)\right\}$$

subject to the capacity constraints in equations (10) and (12).

To describe the outer optimization, let *SP*_*w*_ and *SP*_*s*_ represent the system prices per kW of peak wind and solar energy, respectively. The system price of the combined renewable capacity is then *SP*_*e*_(*s*) = *s* ⋅ *SP*_*w*_ + (1 − *s*) ⋅ *SP*_*s*_. Let *SP*_*h*_ denote the system price per kW of peak power absorption of the PtG plant. In addition to investment costs, the PtG system incurs annual fixed costs, such as insurance and maintenance expenditures. Let *F*_*wi*_ and *F*_*si*_ represent the fixed operating costs per kW of wind and solar capacity in year *i*. The combined fixed costs are then *F*_*ei*_(*s*) = *s* ⋅ *F*_*wi*_ + (1 − *s*) ⋅ *F*_*si*_. We further denote by *F*_*hi*_ the fixed operating costs per kW of the PtG plant in year *i*.

Investment returns are affected by corporate income taxes through the corporate tax rate and the allowable tax shields for debt and depreciation. Let *γ* = (1+*r*)^−1^ represent the discount factor with *r* as the applicable cost of capital. The parameter *r* can be interpreted as the weighted average cost of capital, provided the cost of debt is incorporated on an after-tax basis to reflect the debt tax shield ^45^. To capture the impact of corporate income taxes, we denote by *d*_*i*_≥0 the percentage of the initial capital expenditure that can be deducted as a depreciation charge in year *i* from revenues when calculating taxable income. By construction, ${\sum }_{i=0}^{T}{d}_{i}=1$. In case the productive capacity of the renewables and the PtG plant degrades over time, let *x*_*i*_ denote the effective share of the initial capacity of the overall system that is still productive in year *i*.

Incorporating the inner optimization from equation (13), the outer optimization problem is given by equation (14):

14 $$NPV(A) \equiv 	 {{\max }_{s,\,{k}_{e},\,{k}_{h}}}\left\{(1-\alpha )\cdot {\sum}_{i=1}^{T}{x}_{i}\cdot {\gamma }^{i}\cdot C{M}_{i}(s,{k}_{e},{k}_{h}| A)-{\gamma }^{i}\cdot \left({F}_{ei}(s)\cdot {k}_{e}+{F}_{hi}\cdot {k}_{h}\right)\right. \\ 	 \left.-\left(1-\alpha \cdot {\sum}_{i=0}^{T}{d}_{i}\cdot {\gamma }^{i}\right)\cdot \left(S{P}_{e}(s)\cdot {k}_{e}+S{P}_{h}\cdot {k}_{h}\right)\right\}.$$

Following recent regulatory guidance, the analysis initially assumes that the investment in a PtG system includes renewables that are incremental to the existing renewable energy supply in the market. Thus, *k*_*e*_ = 1 if *k*_*h*_ > 0. Overall, the investor would invest in a PtG system if and only if *NPV*(*A*)≥0. This condition becomes binding when an investment in renewables alone is not economically viable, and the addition of a PtG plant increases the overall net present value but not enough to make it non-negative^46^.

The preceding model structure requires minor modifications if accounting rules *B* or *C* apply. Under rule *D*, investors can choose whether to co-invest in renewables. That is, they can freely choose *k*_*e*_ on the interval [0, 1] for the outer optimization. In addition, they can procure EACs for renewable energy from non-incremental sources on the open market. Since most EACs traded today are not time-specific, we denote by ${v}_{ri}^{\circ }$ the amount of EACs (in kWh) procured in year *i* and by *p*_*ri*_ the price per kWh at which EACs can be bought on the market in year *i*. The total cost of EACs procured in year *i* is thus ${p}_{ri}\cdot {v}_{ri}^{\circ }$. If this term is subtracted from, say, the pre-tax contribution margin of hydrogen production *CM*_*hi*_( ⋅ ) in equation (11), the optimization can proceed as described above.

To evaluate the cost-effectiveness of the policy support, the policy impact of the tax credit is measured as the total hydrogen produced over the life cycle of a PtG system divided by the discounted value of the annual tax credits awarded by the government. With *r* denoting the applicable cost of capital, the policy impact (in kg H_2_ per $) under, say, accounting rule *A* is given by:

15 $$PI(A)\equiv \frac{{\sum }_{i=1}^{T}{\sum }_{t=1}^{m}{q}_{i}(t| A)}{\mathop{\sum }_{i=1}^{T}PT{C}_{hi}(A)\cdot {(1+r)}^{-i}}.$$

### Model Calibration

The model is initially calibrated to reference plants eligible for the production tax credit available under the Inflation Reduction Act in the current economic context of Texas in the US. The data inputs come from multiple sources, including journal articles, technical reports, and industry databases. All data inputs are provided in an Excel file included in the Supplementary Data. An overview of the main cost and operational parameters is provided in Supplementary Note 1.

Our main analysis considers a PtG plant with a PEM electrolyzer. The corresponding system price is based on recent industry data^47,48^ and includes acquisition, project development, and installation costs for both electrolyzer stacks and balance-of-system components. The operating performance follows a constant-power control strategy, in which the electrolyzer adjusts its current to meet power setpoints that can vary flexibly between 5–100% of peak capacity^49^. This abstracts from the technological possibility that PEM electrolyzers could briefly operate above peak capacity and approximates that Megawatt-scale PEM electrolyzers can ramp up or down at a rate of up to 10% of their peak capacity per second^50^. The analysis further presumes a constant conversion efficiency, approximating that PEM electrolyzers attain a near-constant energy consumption beyond a small threshold utilization level^51^.

System prices for wind and solar energy are calculated as the arithmetic averages of the median values provided in recent reports for utility-scale onshore wind by Lazard^52^ and the Lawrence Berkeley National Laboratory^53^, as well as recent reports for utility-scale solar photovoltaic by the National Renewable Energy Laboratory (NREL)^54^ and the Lawrence Berkeley National Laboratory^55,56^. All system prices are expressed in 2024 $US and were adjusted to the price level in Texas using regional price parities from the US Bureau of Economic Analysis^57^.

Hourly capacity factors for wind energy are calculated using the Pluswind dataset^58^. In particular, we selected a location in the MERRA-2 model^59^ that reflects the median value of annual average capacity factors across locations in Texas and across the MERRA-2, HRRR, and ERA-5 data models^59^. Hourly capacity factors for solar energy are calculated using the PySAM package^60^. Similar to the procedure for wind energy, we selected a location in the dataset that reflects the median value of annual average capacity factors across Texas. To generate capacity factors in PySAM, we used the PVWatts v8 module based on typical meteorological year weather data, with key parameters set to 1000 kW_dc_ system capacity, fixed tilt of south-facing (180^∘^ azimuth), and 1.2 DC-to-AC ratio. Solar and meteorological data were sourced from the National Solar Radiation Database^61^, specifically the Physical Solar Model v3.2.2 TMY dataset.

Hourly sales prices for electricity on the wholesale market are calculated based on ERCOT day-ahead prices obtained from Hitachi Energy’s Velocity Suite^62^. To construct a reference year, we calculate a simple price vector where each hourly price is equal to the average across the day-ahead prices observed in Texas between the years 2015–2024 for the corresponding hour:

16 $${p}_{s}(t)=\frac{1}{10}\mathop{\sum }_{i=2015}^{2024}{p}_{si}(t).$$

The resulting price vector in equation (16) reflects a deregulated electricity market with a substantial share of renewable power generation. Based on this vector of hourly sales prices for electricity, we calculate a vector of hourly buying prices for electricity by adding a cost markup for grid surcharges and other retail charges for large-scale industrial customers. We set this cost markup 1.0% higher than the cost markup *δ*_*e*_ incurred for converting dedicated renewable energy to ensure that the optimization algorithm prioritizes the conversion of renewable energy over the conversion of carbon-intensive electricity from the general grid. This adjustment has a negligible effect on the profitability of PtG systems.

Hourly carbon intensity values for general grid electricity are calculated based on the EIA-930 dataset by the Energy Information Administration^63^, using the CO2i_ERCO_D time series available from 2019–2023. Similar to the procedure for hourly electricity sales prices, we calculate a reference vector where each hourly carbon intensity is equal to the average across the carbon intensity levels observed in Texas between the years 2019–2023 for the corresponding hour:

17 $${CI}_{e}(t)=\frac{1}{5} \mathop{\sum}_{i=2019}^{2023}C{I}_{ei}(t).$$

Like for electricity prices, the resulting carbon intensity vector in equation (17) reflects a deregulated electricity market with a substantial share of renewable power generation.

The economic model is implemented in Python using optimization packages from Gurobi^64^ and SciPy^65^. In particular, we use a Mixed-Integer Linear Programming approach from Gurobi for the inner optimization to capture the stepwise granting of the hydrogen production tax credit in equation (4). We then use a differential evolution algorithm from SciPy for the outer optimization. To mitigate computational costs, the numerical optimization initially assumes that the hourly distribution of electricity prices, capacity factors, and carbon intensity levels of general grid electricity remains constant over the lifetime of a PtG system. Since production tax credits are only available for the first ten years of the investment, we run the inner optimization for two representative years: the first year of operation with tax credits and the eleventh year of operation without tax credits.

We run the inner optimization of the specifications reported in the main body of the paper for 8760 h. Since this takes considerable computational time and each sensitivity analysis reported in Supplementary Notes 4–13 requires several runs of the optimization program, we run the inner optimization for these calculations for the same 1000 randomly selected hours instead of a full year. Differences between the results for 1000 h and a full year are small, as shown in Supplementary Note 3.

## Supplementary information

Supplementary Information

Description of Additional Supplementary Files

Supplementary Data 1

Transparent Peer Review file

## Data Availability

The data used in this study are referenced in the main body of the paper and the Supplementary Information. Data that generated the plots in the paper are provided in an Excel file available as part of the Supplementary Data. Figures are provided on Figshare (10.6084/m9.figshare.29477357).

## Code availability

Computational code is available on GitHub.
