# Supplementary material for: How carbon accounting rules shape incentives for hydrogen production
Source: Nat Commun. 2026 Aug 10;17:7260. doi: 10.1038/s41467-026-75473-z (PMC13457867; doi:10.1038/s41467-026-75473-z)
Supplement: Supplementary file 1 [file 41467_2026_75473_MOESM1_ESM.pdf]

Supplementary Information

How Carbon Accounting Rules Shape Incentives for Hydrogen Production

Gunther Glenk[1,2,*], **Philip Holler**[1,*], and **Stefan Reichelstein**[1,3,4]

[1]Business School, University of Mannheim, Mannheim, Germany

[2]Center for Energy and Environmental Policy Research, Massachusetts Institute of Technology, Cambridge, MA, United States

[3]Graduate School of Business, Stanford University, Stanford, CA, United States

[4]ZEW – Leibniz Centre for European Economic Research, Mannheim, Germany

[*]Correspondence: glenk@uni-mannheim.de; philip.holler@uni-mannheim.de

Supplementary List of Contents

Supplementary Note 1. Main Input Parameters . . . 3
Supplementary Table 1. Main cost and operational parameters . . . 3
Supplementary Note 2. Hourly Operation of PtG Systems . . . 4
Supplementary Figure 1. Hourly operation at a hydrogen price of $1.5 per kilogram . . . 5
Supplementary Figure 2. Hourly operation at a hydrogen price of $2.5 per kilogram . . . 6
Supplementary Figure 3. Hourly operation at a hydrogen price of $3.5 per kilogram . . . 7
Supplementary Note 3. Temporal Resolution of the Optimization Program . . . 8
Supplementary Figure 4. Life-cycle performance under different temporal resolutions . . . 9
Supplementary Note 4. Location-Based Methods . . . 10
Supplementary Figure 5. Life-cycle performance under location-based methods . . . 10
Supplementary Note 5. Reduced Production Tax Credits . . . 11
Supplementary Figure 6. Life-cycle performance under reduced production tax credits . . . 12
Supplementary Note 6. Changes in the Carbon Intensity of Electricity . . . 13
Supplementary Figure 7. Life-cycle performance under lower future carbon intensity levels of general grid electricity . . . 14
Supplementary Note 7. Changes in Electricity Prices . . . 15
Supplementary Figure 8. Life-cycle performance under changes in future electricity prices 17
Supplementary Note 8. Changes in Electricity Prices and Carbon Intensity . . . 18
Supplementary Figure 9. Life-cycle performance under changes in future electricity prices and carbon intensity levels . . . 20
Supplementary Note 9. Other Geographic Regions . . . 21
Supplementary Table 2. Main changes in input parameters . . . 21
Supplementary Figure 10. Life-cycle performance in different states . . . 24
Supplementary Note 10. Alternative Degradation Rates . . . 25
Supplementary Figure 11. Life-cycle performance under different degradation rates . . . 26
Supplementary Note 11. Alkaline Electrolyzers . . . 27
Supplementary Table 3. Changes in input parameters . . . 27
Supplementary Figure 12. Life-cycle performance of systems with alkaline electrolyzers . . 28
Supplementary Note 12. Alternative Electrolyzer Costs . . . 29
Supplementary Table 4. Changes in cost parameters . . . 29
Supplementary Figure 13. Life-cycle performance of PtG systems with PEM electrolyzers at lower capacity-related costs . . . 30
Supplementary Figure 14. Life-cycle performance of PtG systems with alkaline electrolyzers at lower capacity-related costs . . . 31

Supplementary Note 13. Marginal Emission Rates . . . 32
Supplementary Figure 15. Life-cycle performance under marginal emission rates . . . 33
Supplementary References . . . 34

Supplementary Note 1. Main Input Parameters

Supplementary Table 1. Main cost and operational parameters.

in 2024 $US	Unit	Value	Sources
**General Parameters**			
Economic lifetime, T	years	20	[1]
Cost of capital, r	%	8.00	
Tax rate, α	%	21	[2]
Depreciation method, d	–	3*	
Degradation factor, x	%/yr	1.00	[3]
**Wind Energy**			
System price, SP_w	$/kW	1,609.00	[4]
Fixed operating cost, F_{wi}	$/kW	12.840	[5]
Average capacity factor, CF_{wi}	%	40.53	[6]
Production tax credit, PTC_{ei}	$/kWh	0.015	[7]
**Solar Energy**			
System price, SP_s	$/kW	1,133.00	[4]
Fixed operating cost, F_{si}	$/kW	5.133	[5]
Average capacity factor, CF_{si}	%	25.30	[6]
Production tax credit, PTC_{ei}	$/kWh	0.015	[7]
**Power-to-Gas**			
System price, SP_h	$/kW	1,500.00	[8]
Fixed operating cost, F_{hi}	$/kW	45.00	[3]
Variable operating cost, w_h	$/kg	0.10	[9]
Conversion rate, η	kg/kWh	0.0189	[9]
**Market Prices & Carbon Intensity**			
Avg. electricity selling price, p_{si}	$/kWh	0.04677	[5]
Avg. electricity buying price, p_{bi}	$/kWh	0.05473	[5]
Markup on renewable energy, δ_e	$/kWh	0.0079	[9]
Avg. carbon intensity of grid, CI_e	kg CO_2e/kWh	0.3862	[10]

*3: 20-year 150%-declining-balance depreciation schedule.
Sources: [1] [1], [2] [2], [3] [3;4], [4] [5–8], [5] [9], [6] Own analysis at `https://bit.ly/4pzmQOG`, [7] [10], [8] [11], [9] [12], [10] [13].

Supplementary Note 2. Hourly Operation of PtG Systems

To illustrate the hourly operation of Power-to-Gas (PtG) systems, Supplementary Figures 1, 2, and 3 show the operation of PtG systems at hydrogen prices of \$1.5, \$2.5, and \$3.5 per kg, respectively. Under accounting rule A, PtG systems typically convert almost only renewable electricity. That is, they procure only small amounts of general grid electricity so as not to exceed an hourly carbon intensity of 0.45 kg CO_2e per kg H_2, the threshold for the highest tax credit (see equation (4) of the main text). As hydrogen prices rise, however, investors are more often incentivized to forgo the tax credit in favor of converting general grid electricity at full capacity. This effect occurs mostly when renewable power generation and electricity market prices are sufficiently low.

Under accounting rule B, PtG systems seek to fully utilize capacity without exceeding an annual average carbon intensity of 0.45 kg CO_2e per kg H_2. As a result, they convert grid electricity only during hours of sufficiently low market prices and carbon intensity levels. As hydrogen prices rise, PtG systems are incentivized to increase the capacity of electrolyzers, k_h, and the relative share of wind energy capacity, s. This applies under both accounting rules A and B.

Under accounting rules C and D, PtG systems are incentivized to fully utilize capacity during most hours of the year. This reflects the flexibility resulting from the annual matching of electricity generation and consumption. Yet, the total amount of electricity converted to hydrogen (and the capacity size of the PtG unit) is substantially lower under rule C than rule D. This is mainly because of the natural limit imposed by the amount of self-generated renewable energy under rule C.

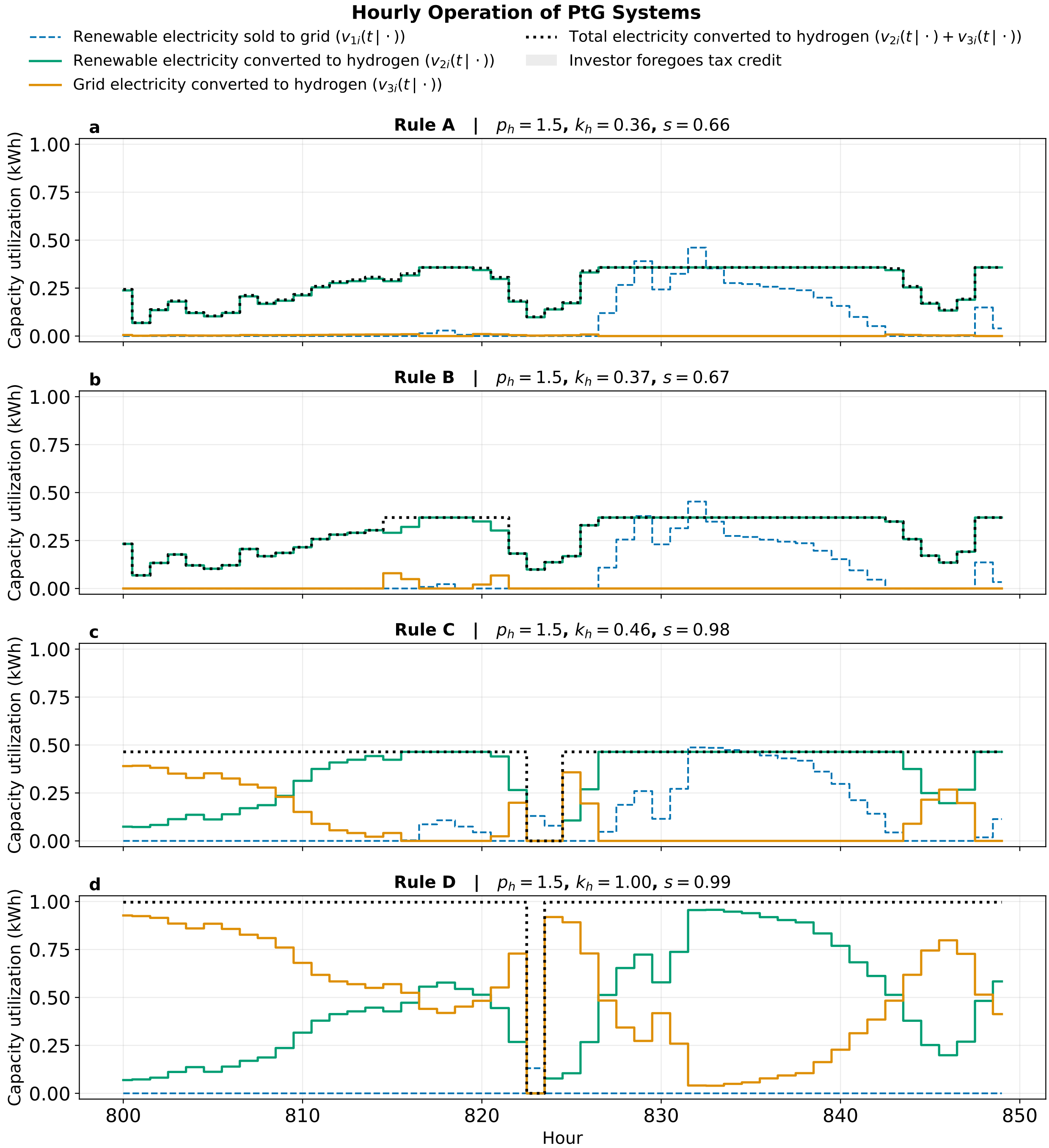

Supplementary Figure 1. Hourly operation of PtG systems. This figure shows the hourly operation of PtG systems for a selection of hours at a hydrogen price of \$1.5 per kg.

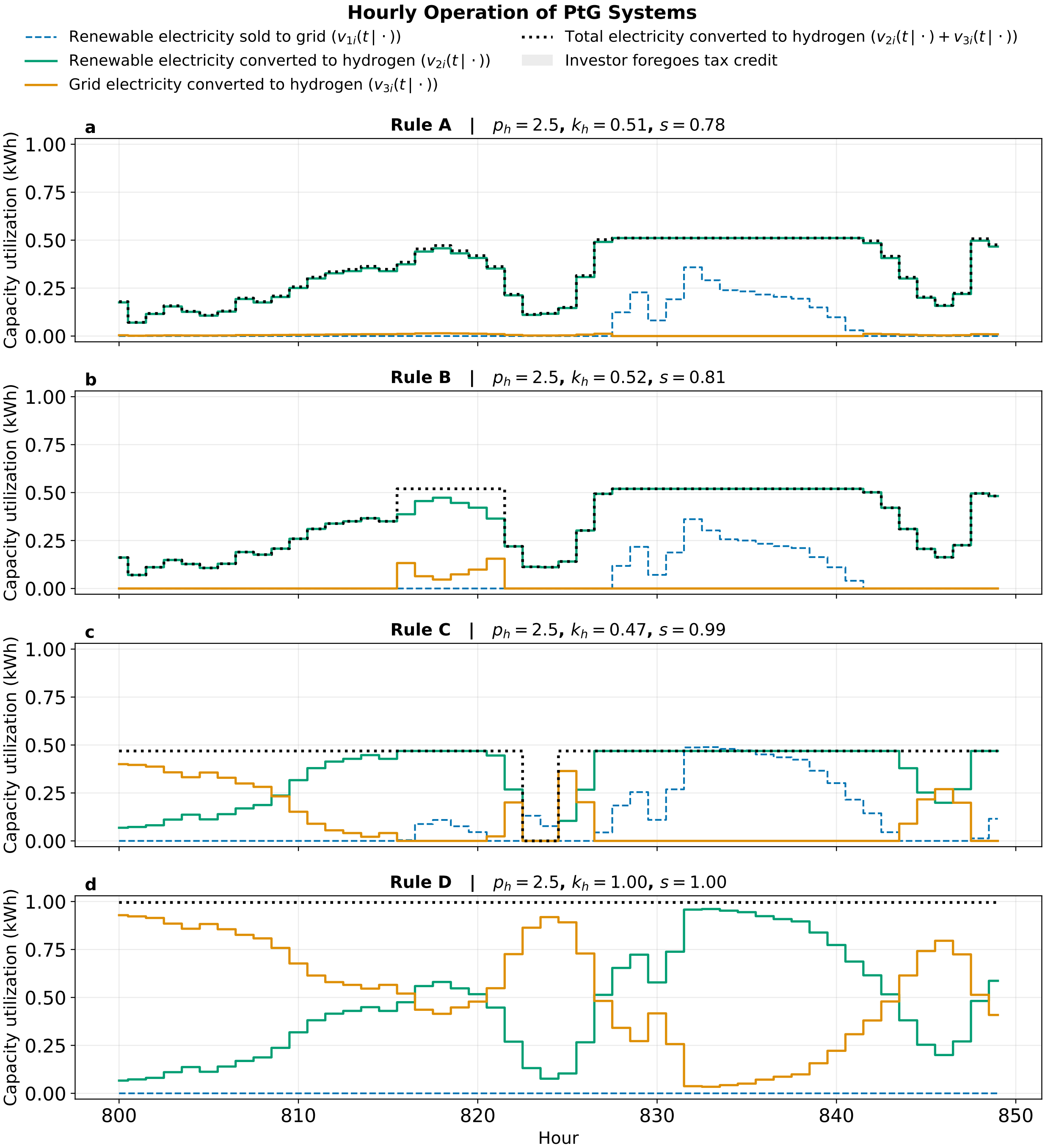

Supplementary Figure 2. Hourly operation of PtG systems. This figure shows the hourly operation of PtG systems for a selection of hours at a hydrogen price of \$2.5 per kg.

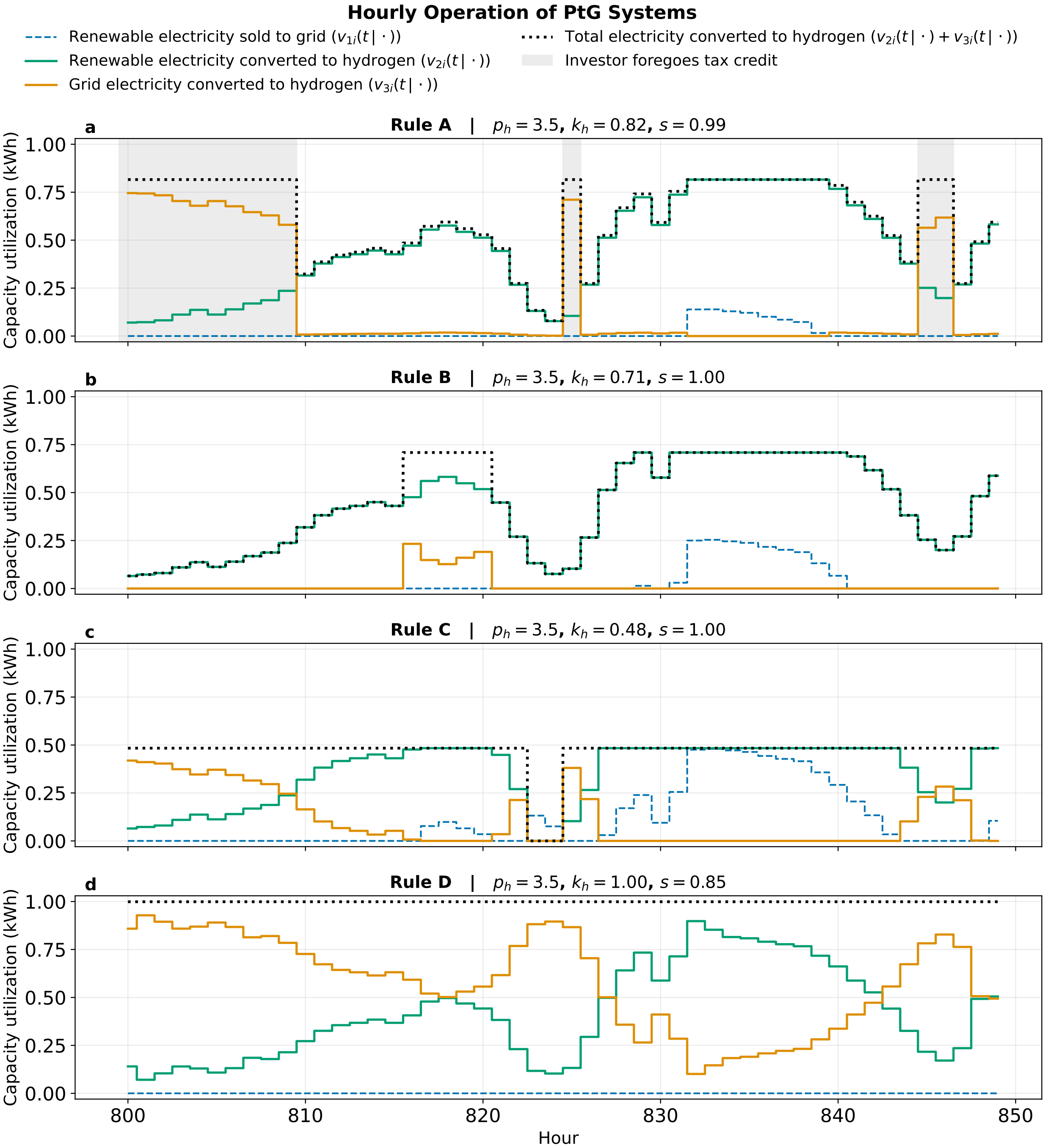

Supplementary Figure 3. Hourly operation of PtG systems. This figure shows the hourly operation of PtG systems for a selection of hours at a hydrogen price of \$3.5 per kg.

Supplementary Note 3. Temporal Resolution of the Optimization Program

We run the optimization program for the specification reported in the main body of the paper at a temporal resolution of 8,760 hours. Because this is computationally intensive and each sensitivity analysis requires several runs of the optimization program, we run the inner optimization for the sensitivity calculations using a subset of 1,000 randomly selected hours rather than the full year.

To test whether the random sampling introduces bias, we here compare the results for the main specification using the subset and the full year. As Supplementary Figure 4 shows, the profitability of PtG systems is slightly higher for the subset, though differences are small. The life-cycle average carbon intensity and the policy impact of the production tax credits are nearly identical under both temporal resolutions.

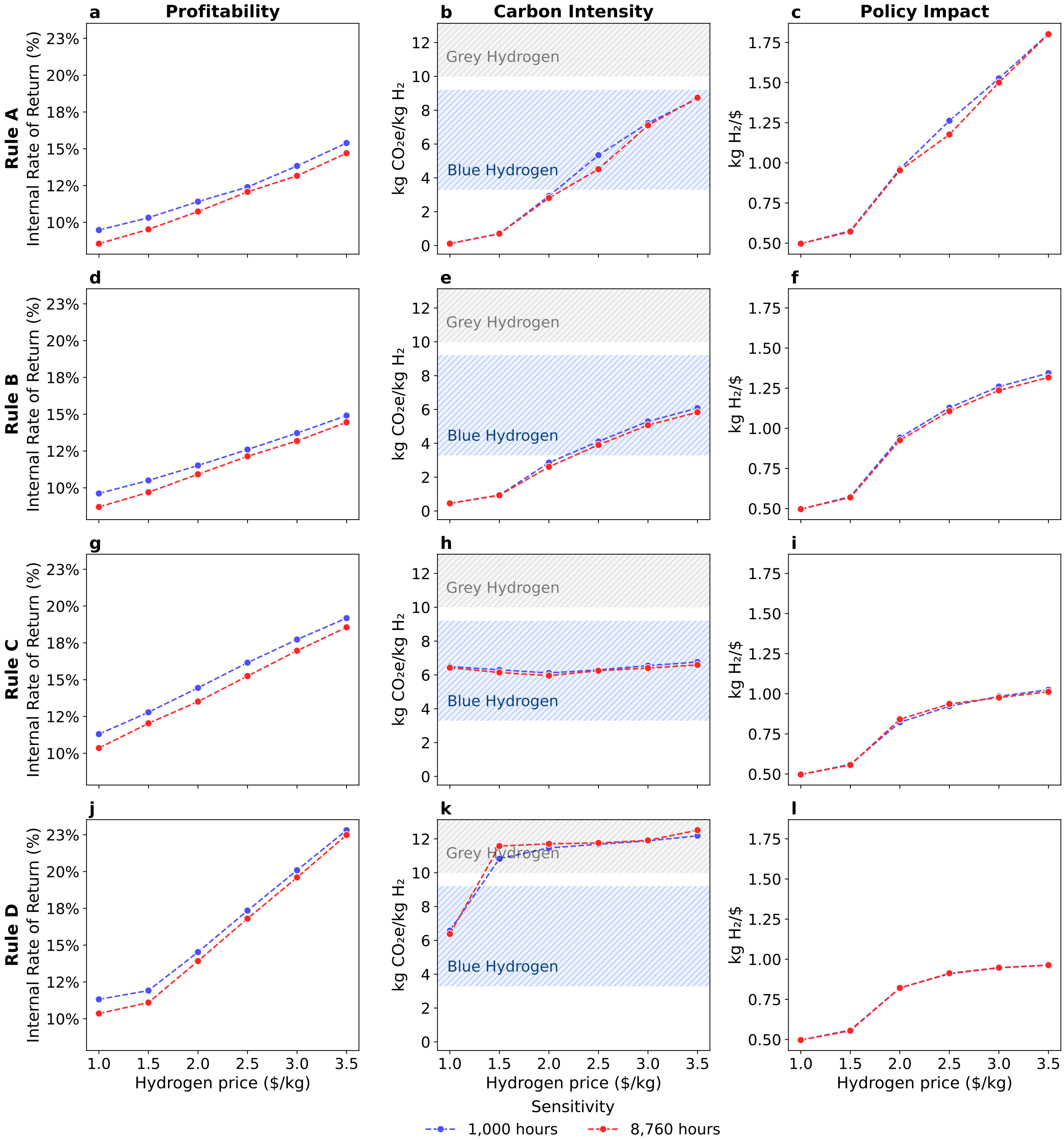

Supplementary Figure 4. Life-cycle performance under different temporal resolutions. This figure shows the impact of the temporal resolution of the optimization program on (**a, d, g, j**) the profitability of PtG systems, (**b, e, h, k**) the life-cycle average carbon intensity of hydrogen, and (**c, f, i, l**) the policy impact of the production tax credit, given hydrogen prices between $1.0 and $3.5 per kg. Dots show the point estimates at specific hydrogen prices, while dashed lines interpolate between them for illustration.

Supplementary Note 4. Location-Based Methods

To examine the impact of location-based methods, initially assume that investors can co-locate renewables with the PtG plant and find the same wind and solar resources as before. Yet, note that areas near hydrogen customers may have less space or weaker wind and solar irradiation. Due to the co-location, PtG plants now incur no cost markup for grid surcharges and other retail charges for renewable energy converted to hydrogen (i.e., $\delta_e = 0$). The analysis focuses on accounting rules A, B, and C, since rule D reflects market-based methods by construction. Let the location-based variants of these rules be denoted by A', B', and C'.

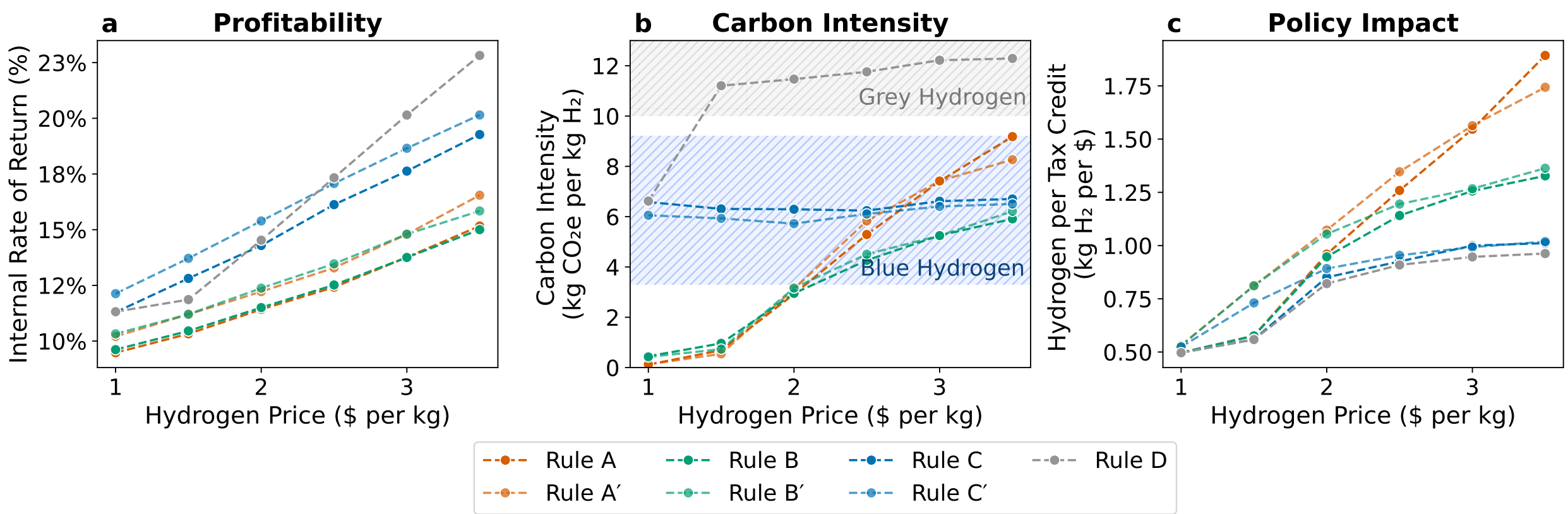

Supplementary Figure 5. Life-cycle performance under location-based methods. This figure shows the impact of location-based methods on (**a**) the profitability of PtG systems, (**b**) the life-cycle average carbon intensity of hydrogen, and (**c**) the policy impact of the production tax credit, given hydrogen prices between \$1.0 and \$3.5 per kg. Dots show the point estimates at specific hydrogen prices, while dashed lines interpolate between them for illustration.

Supplementary Figure 5 shows our estimates for the implications of location-based methods. We find that the performance of PtG systems under these methods is largely parallel to the corresponding one shown in Fig. 4 of the main text. In particular, we find that the profitability of PtG systems is slightly higher, the life-cycle average carbon intensity of hydrogen is slightly lower, and the policy impact is slightly higher. These differences are primarily due to the avoidance of grid charges on generated renewable energy under location-based methods. As a result, investors build relatively more renewable energy capacity and convert relatively more renewable energy, especially after the tax credit period.

Supplementary Note 5. Reduced Production Tax Credits

To examine the effect of reduced production tax credits, we multiply the function $f(\cdot)$ specified in equation (4) of the main text by the scalar λ and repeat the analysis for $\lambda = \{0.9, 0.8, 0.7\}$. These values reflect a reduction of the tax credits by 10, 20, and 30%.

Supplementary Figure 6 shows our estimates for the implications of reduced production tax credits. As one might expect, we find that the profitability of PtG systems declines as production tax credits decline. The reduction of internal rates of return, however, is mitigated by the fact that investors are incentivized to build smaller PtG plants. As a result, the profitability of PtG system still ranges between 8.0–15.0% under accounting rules A and B, while it is significantly higher under accounting rules C and D in most variations.

As for emissions, we find that the life-cycle average carbon intensity of hydrogen is fairly insensitive to the changes in production tax credits. One exception is that, under accounting rule D, the jump in the life-cycle average carbon intensity now occurs at higher hydrogen prices. This reflects that, below a certain hydrogen price, it is economically unattractive for investors to procure any Energy Attribute Certificates (EACs) for renewable energy on the open market.

We further find that the policy impact of the tax credits increases as the tax credits are reduced. In other words, the policy program becomes more effective in incentivizing electrolytic hydrogen production. This finding reflects that investors would build smaller PtG plants and produce less hydrogen. Yet, this reduction is outweighed by the reduction in tax credits paid.

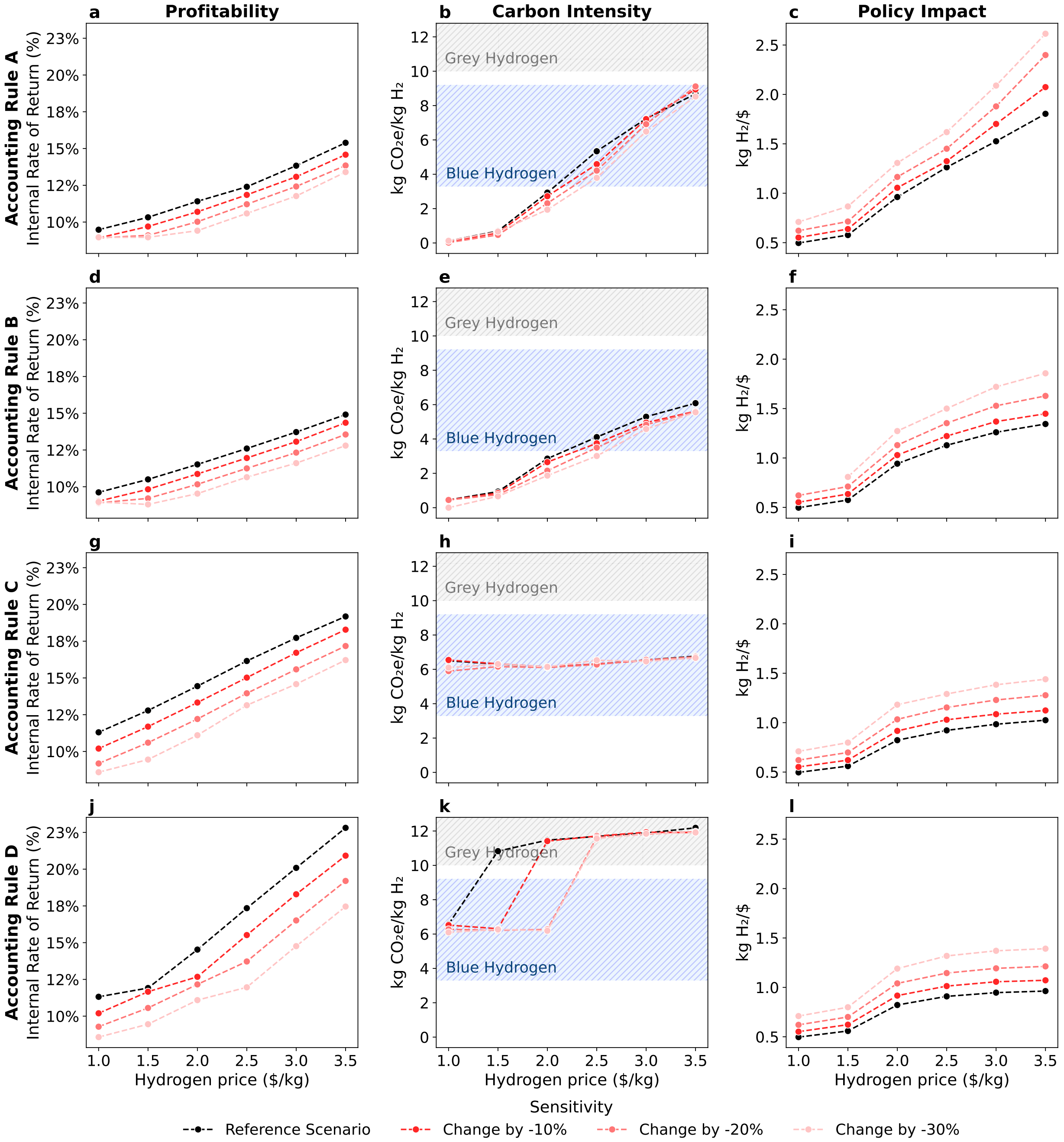

Supplementary Figure 6. Life-cycle performance under reduced production tax credits. This figure shows the impact of reduced production tax credits on (**a, d, g, j**) the profitability of PtG systems, (**b, e, h, k**) the life-cycle average carbon intensity of hydrogen, and (**c, f, i, l**) the policy impact of the production tax credit, given hydrogen prices between \$1.0 and \$3.5 per kg. Dots show the point estimates at specific hydrogen prices, while dashed lines interpolate between them for illustration.

Supplementary Note 6. Changes in the Carbon Intensity of Electricity

This section examines the impact of lower future carbon intensity levels of general grid electricity to reflect higher shares of renewable power generation in the future. Recall from the Methods of the main text that, to mitigate computational costs, our numerical calibration runs the inner optimization for two representative years: the first year of operation with tax credits and the eleventh year of operation without tax credits.

To examine the impact of lower future carbon intensity levels, we multiply the reference vector for the hourly carbon intensity of general grid electricity by a reduction factor and use this adjusted vector only for the second inner optimization (i.e., the eleventh year of operation). The analysis considers three alternative projections for the carbon intensity of general grid electricity, as provided by the National Renewable Energy Laboratory (NREL)[14]. The central scenario projects a reduction of 64% by 2035, while the lower and upper scenarios project reductions of 54% and 75%.

Supplementary Figure 7 shows our estimates for the implications of lower future carbon intensity levels of general grid electricity. As one might expect, we find that the profitability of PtG systems and the policy impact of the tax credits remain unchanged. This is because the reduction in carbon intensity levels only applies for the period after the tax credits. In terms of emissions, however, we find that the life-cycle average carbon intensity of hydrogen declines significantly as general grid electricity becomes less carbon-intensive. In particular, we find that the life-cycle average carbon intensity levels under accounting rule B are now below those for blue hydrogen at all hydrogen prices considered. Under rule D, they are now below those for grey hydrogen and close to upper estimates for blue hydrogen at all hydrogen prices considered.

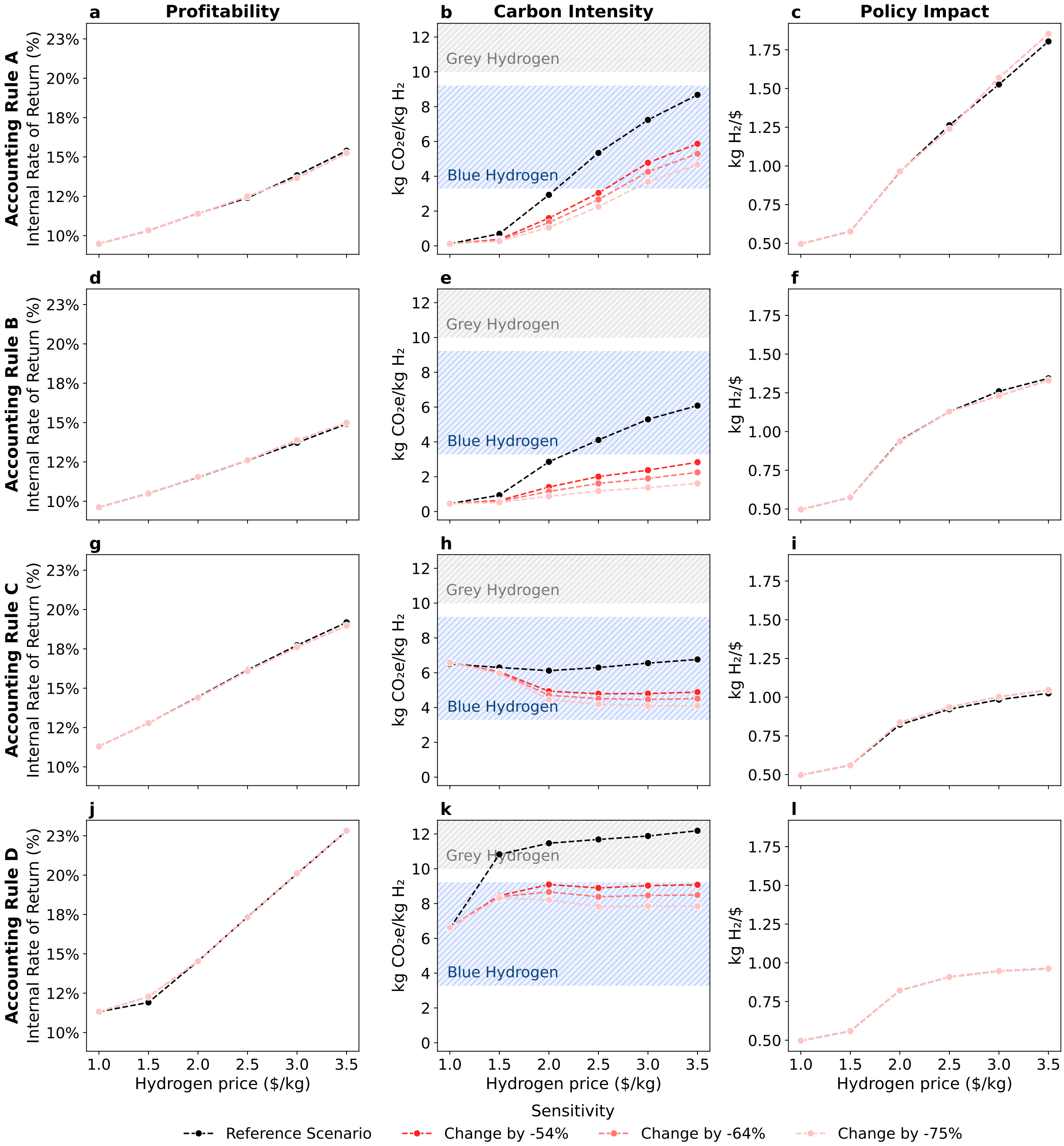

Supplementary Figure 7. Life-cycle performance under lower future carbon intensity levels of general grid electricity. This figure shows the impact of lower future carbon intensity levels of general grid electricity on (**a, d, g, j**) the profitability of PtG systems, (**b, e, h, k**) the life-cycle average carbon intensity of hydrogen, and (**c, f, i, l**) the policy impact of the production tax credit, given hydrogen prices between \$1.0 and \$3.5 per kg. Dots show the point estimates at specific hydrogen prices, while dashed lines interpolate between them for illustration.

Supplementary Note 7. Changes in Electricity Prices

In this section, we examine the implications of changes in the average (α) and variance (β) of future electricity prices to reflect the growing share of renewable power generation. To that end, let $\mu(t)$ denote the multiplicative deviation factor of electricity selling prices given by equation (S1):

$$p_s(t) \equiv \mu(t) \cdot \frac{1}{m} \sum_{t=1}^{m} p_s(t). \tag{S1}$$

By construction, equation (S2) holds:

$$\sum_{t=1}^{m} \mu(t) = 1. \tag{S2}$$

Furthermore, let α denote the relative change in the annual average of electricity prices and β the relative change in the hourly variation of electricity prices during hours where prices are above average. In addition, we calculate the corresponding change in the hourly variation of electricity prices during hours where prices are below average, denoted by $\hat{\beta}$, such that the adjusted annual average remains unchanged. Thus, the adjusted electricity price in a particular hour is given by equation (S3):

$$\hat{p}_s(t) = \begin{cases} \beta \cdot \mu(t) \cdot \alpha \cdot \frac{1}{m} \sum_{t=1}^{m} p_s(t) & \text{for } t \text{, where } \mu(t) \geq 1, \\ \hat{\beta} \cdot \mu(t) \cdot \alpha \cdot \frac{1}{m} \sum_{t=1}^{m} p_s(t) & \text{for } t \text{, where } \mu(t) < 1, \end{cases} \tag{S3}$$

where $\hat{\beta}$ is calculated such that equation (S4) holds:

$$\frac{1}{m} \sum_{t=1}^{m} \hat{p}_s(t) = \alpha \cdot \frac{1}{m} \sum_{t=1}^{m} p_s(t). \tag{S4}$$

The vector for hourly electricity buying prices, $p_b(t)$, is adjusted in direct analogy to that. Note that our sensitivity analysis does not aim to develop a new model for future electricity prices. Instead, it examines the impact of different electricity price distributions on the financial and emission performance of PtG systems, given accounting rules A through D. In particular, the analysis examines permutations of simultaneous changes for α and β up to $\pm 30\%$, due to computational cost in steps of 15%. We use the adjusted electricity price vectors only for the second inner optimization (i.e., the eleventh year of operation onward).

Supplementary Figure 8 shows our estimates for the implications of changes in electricity

prices. We find that, under accounting rules A through C, the profitability of PtG systems slightly increases (decreases) as the average of electricity prices increases (decreases), whereas decreases (increases) in the variance of electricity prices mitigate this effect, especially at higher hydrogen prices. This mainly reflects that at lower hydrogen prices, the generated renewable energy can be sold at higher (lower) market prices. Under rule D, the profitability of PtG systems appears to increase as the average of electricity prices either increases or decreases. We attribute this effect to the following observation: When the average of electricity prices declines, PtG systems can buy general grid electricity at lower market prices. When the average of electricity prices rises, however, the generated renewable energy can be sold at higher market prices.

Regarding emissions, we find that, under accounting rules A and B, the life-cycle average carbon intensity of hydrogen tends to decline (strongly increase) as the average of electricity prices increases (decreases). This reflects that PtG systems convert less (significantly more) general grid electricity. Under accounting rules C and D, life-cycle average carbon intensity levels of hydrogen remain almost unaffected when the average of electricity prices increases. This is mainly due to the natural limit on the amount of general grid electricity that can be converted, imposed by the generated renewable energy. Yet, the life-cycle average carbon intensity levels of hydrogen increase significantly when average prices decline. PtG systems, at all changes in the average of electricity prices considered, now produce at almost full capacity after the tax credit expires, resulting in relatively high and constant life-cycle average carbon intensity levels at higher hydrogen prices.

As for the policy impact, we generally find that lower averages and higher variances in electricity prices lead to a greater policy impact. While scenarios with smaller changes in both parameters remain closer to the reference scenario, scenarios with larger changes have a significant impact on the life-cycle production of hydrogen per $ of tax credit. The largest impact of the differences in the average (α) and variance (β) of electricity prices occurs under accounting rule A. This is due to the operational flexibility investors have under this rule. For rules B through D, some convergence of the policy impact occurs with increasing hydrogen prices.

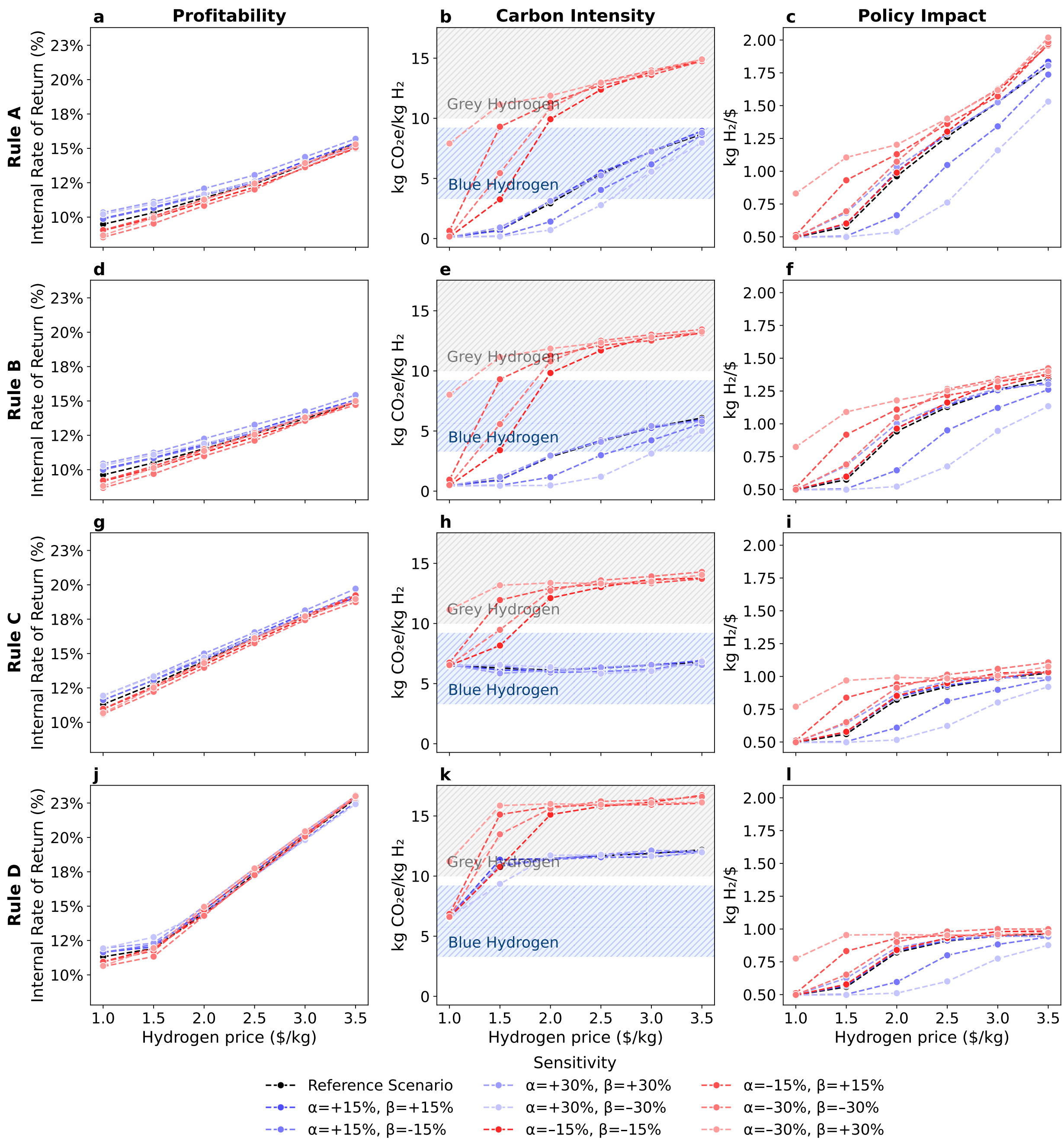

Supplementary Figure 8. Life-cycle performance under changes in future electricity prices. This figure shows the impact of simultaneous changes in the average (α) and variance (β) of future electricity prices on (**a, d, g, j**) the profitability of PtG systems, (**b, e, h, k**) the life-cycle average carbon intensity of hydrogen, and (**c, f, i, l**) the policy impact of the production tax credit, given hydrogen prices between \$1.0 and \$3.5 per kg. Dots show the point estimates at specific hydrogen prices, while dashed lines interpolate between them for illustration.

Supplementary Note 8. Changes in Electricity Prices and Carbon Intensity

With the growing share of renewable power generation, it is widely expected that prices and carbon intensity levels of grid electricity will change simultaneously. To examine the joint effect of both changes, we combine the adjustments in the preceding two subsections. In particular, we consider the central scenario for the changes in the carbon intensity of general grid electricity, which projects a reduction of 64% by 2035. In addition, we examine permutations of simultaneous changes for α and β up to $\pm 30\%$, due to computational cost in steps of 15%. We again use the adjusted vectors for the electricity prices and the carbon intensity levels of grid electricity only for the second inner optimization (i.e., the eleventh year of operation onward).

In addition to these scenarios, we also analyze a forward-looking scenario for hourly prices and grid emission rates using the Cambium dataset from NREL[15]. Cambium combines a capacity-expansion model (ReEDS) with an hourly production-cost model (PLEXOS) to project future grid operations, including hourly cost and emission metrics under alternative transition pathways[16]. Using projections from the mid-case scenario, we replace the vector of hourly carbon intensity levels for the second inner optimization (i.e., the eleventh year of operation onward) with the projected hourly average emission rates from Cambium. We also replace the vector of hourly wholesale electricity prices for the second inner optimization with the projected hourly marginal system costs (i.e., the parameter total_cost_busbar) from Cambium. This measure captures hourly totals of the short-run marginal power generation costs and markups for capacity-related costs.

Supplementary Figure 9 shows the projections for simultaneous changes in electricity prices and the carbon intensity of general grid electricity. Consistent with the preceding results, we find that the profitability of PtG systems and the policy impact of the tax credits remain nearly unchanged relative to Supplementary Figure 8. This is primarily because the reduction in carbon intensity applies only after the tax credit period. In terms of emissions, we find that the life-cycle carbon intensity of hydrogen is substantially lower than in Supplementary Figure 8, reflecting the lower future carbon intensity levels of general grid electricity. This downward shift is particularly pronounced when the average electricity price decreases, as PtG systems procure more general grid electricity relative to the scenarios where the average electricity price increases.

The results based on the Cambium dataset are generally close to those based on the projections for most scenarios considered. In particular, the results for the profitability of

PtG Systems and the life-cycle average carbon intensity of hydrogen based on Cambium form a close lower envelope to those based on the projections. This partly reflects that the projected average decarbonization of the grid by 2035 is lower than in the projections, resulting in systematically lower average emission intensity levels. This also reflects that the projected marginal system costs in Cambium decline on average while their hourly variability increases, providing incentives for flexible operation without materially altering profitability outcomes.

Overall, the life-cycle average carbon intensity levels under accounting rule A are now close to lower and middle estimates for blue hydrogen at higher hydrogen prices. Under rule B, they are now close to (below) lower estimates for blue hydrogen when the average electricity price decreases (increases), across all hydrogen prices considered. Under rule C, they remain close to the reference scenario when the average electricity price decreases, across all hydrogen prices considered. This reflects that the changes in future electricity prices and carbon intensity levels of general grid electricity effectively offset one another. Under rule D, the life-cycle average carbon intensity levels are now close to upper estimates for blue hydrogen for all hydrogen prices considered.

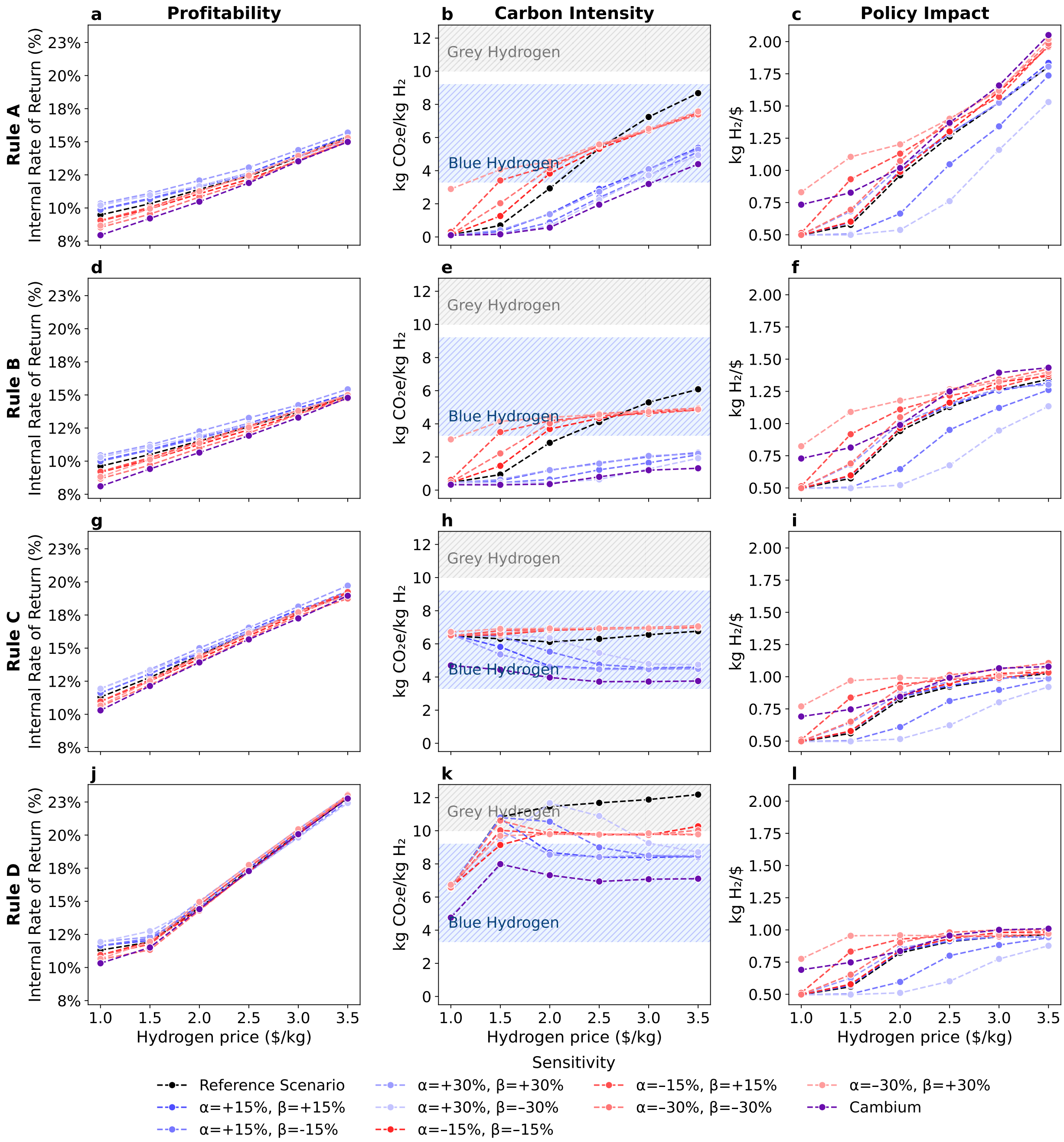

Supplementary Figure 9. Life-cycle performance under changes in future electricity prices and carbon intensity levels of general grid electricity. This figure shows the impact of simultaneous changes in the average (α) and variance (β) of electricity prices on (**a, d, g, j**) the profitability of PtG systems, (**b, e, h, k**) the life-cycle average carbon intensity of hydrogen, and (**c, f, i, l**) the policy impact of the production tax credit, given hydrogen prices between \$1.0 and \$3.5 per kg. Dots show the point estimates at specific hydrogen prices, while dashed lines interpolate between them for illustration.

Supplementary Note 9. Other Geographic Regions

Differences in the technological and economic environments of different states and regions will affect the life-cycle performance of PtG systems. To examine these variations, we rerun the analysis for California, Iowa, and New York. These states, and the corresponding electricity grid systems, differ considerably in terms of their current power generation mix, wholesale market prices, carbon intensity levels of grid electricity, and the availability of wind and solar resources.

California is characterized by high solar irradiation, substantial solar and natural gas power generation, a relatively low carbon intensity of general grid electricity, and frequent periods of low or negative wholesale electricity prices during the day. Iowa has abundant wind resources and obtains a sizable share of its electricity from wind energy. Yet, it also still relies substantially on coal and natural gas power generation, resulting in a relatively high carbon intensity of general grid electricity. New York generates most of its electricity from natural gas in combination with nuclear and hydro-electric generation, resulting in carbon intensity levels of general grid electricity that are comparable to those in California. Wind and solar resources in New York are both limited relative to the other two states.

To isolate the impact of alternative carbon accounting rules, we run the analysis using the main specification described in the Methods of the main text. Changes in future prices and carbon intensity levels of general grid electricity would affect the results in direct analogy to the results in Supplementary Notes 6–8. For each state, we substitute the respective hourly vectors for (i) electricity selling and buying prices, (ii) capacity factors of wind and solar energy, and (iii) carbon intensity levels of general grid electricity. All other cost and operational parameters remain unchanged from the initial calibration to Texas. Supplementary Table 2 lists the main changes in input parameters, while details are provided in the Excel file available as part of the Supplementary Data.

Supplementary Table 2. Main changes in input parameters.

in 2024 $US	**Texas**	**California**	**Iowa**	**New York**
Avg. capacity factor of wind, CF_{wi} (%)	40.53	15.08	38.19	19.22
Avg. capacity factor of solar, CF_{si} (%)	25.30	28.45	22.42	19.12
Avg. electricity selling price, p_{si} ($/kWh)	0.04677	0.04516	0.03556	0.02801
Avg. electricity buying price, p_{bi} ($/kWh)	0.05473	0.05312	0.04352	0.03596
Avg. carbon intensity of grid, CI_{ei} (kg CO_2e/kWh)	0.3862	0.2548	0.4948	0.2465

Supplementary Figure 10 shows our estimates for the life-cycle performance of PtG systems across states. The findings suggest that the simultaneous changes in input parameters

associated with each state change the absolute profitability of PtG systems, life-cycle average carbon intensity of electrolytic hydrogen, and policy impact of the tax credit. Yet, the main conclusions about the relative impact of alternative carbon accounting rules are consistent across states.

In particular, we find that accounting rules A and B yield sufficient profitability of PtG systems today and, in each state, induce a nearly identical profitability distribution across the hydrogen prices considered. An exception to these findings is New York: limited wind and solar resources would lead to negative internal rates of return at hydrogen prices below \$2.0 per kg and to a notably higher profitability under rule A than under rule B at higher hydrogen prices. We also find that accounting rules C and D induce substantially higher profitability of PtG systems than the two hourly accounting rules. Furthermore, the profitability of PtG systems grows more sharply as hydrogen prices rise if non-incremental renewable energy is permitted and investors can exploit the effective price arbitrage between EACs and tax credits under rule D. This effect is particularly pronounced in New York. Overall, the calculations project the highest profitability for PtG systems in Texas and the lowest for systems in New York (except under rule D), while California and Iowa exhibit similar profitability levels that fall between the other two states.

In terms of emissions, the calculations project that accounting rules A and B yield a lower life-cycle average carbon intensity than conventional hydrogen production, but not necessarily much lower. For higher hydrogen prices, carbon intensity levels for electrolytic hydrogen tend be comparable to those for blue hydrogen. They also tend to be higher under rule A than under rule B, as PtG systems under rule A are incentivized to forgo the tax credit in favor of a higher conversion of general grid electricity. Under accounting rules C and D, life-cycle average carbon intensity levels consistently fall in the range of hydrogen production from natural gas. Rule C yields relatively stable carbon intensity levels for electrolytic hydrogen across hydrogen prices and states that fall right in the middle of estimates for blue hydrogen. Rule D yields carbon intensity levels for electrolytic hydrogen that are close to lower estimates for grey hydrogen across most hydrogen prices and states. Overall, the calculations estimate that the life-cycle average carbon intensity of hydrogen is relatively similar in Texas, California, and New York, while Iowa exhibits slightly lower values under rules A through C and notably higher values under rule D.

As for the policy impact of tax credits, we find that a tax credit of \$3.0 per kg induces the production of more than 1.0 kg of hydrogen for all hydrogen prices, all four carbon accounting rules, and all four states considered in this analysis. We also find that, for hydrogen prices up to \$1.5 per kg, the policy impact is nearly identical across all accounting rules and states. For prices above \$1.5 per kg, the policy impact increases under all accounting rules in all

states, whereas it increases more sharply under accounting rules A and B. This sharper increase is fairly similar in Texas and California, while it is substantially lower (higher) in Iowa (New York). Under accounting rules C and D, the increase in the policy impact is nearly identical across all states considered.

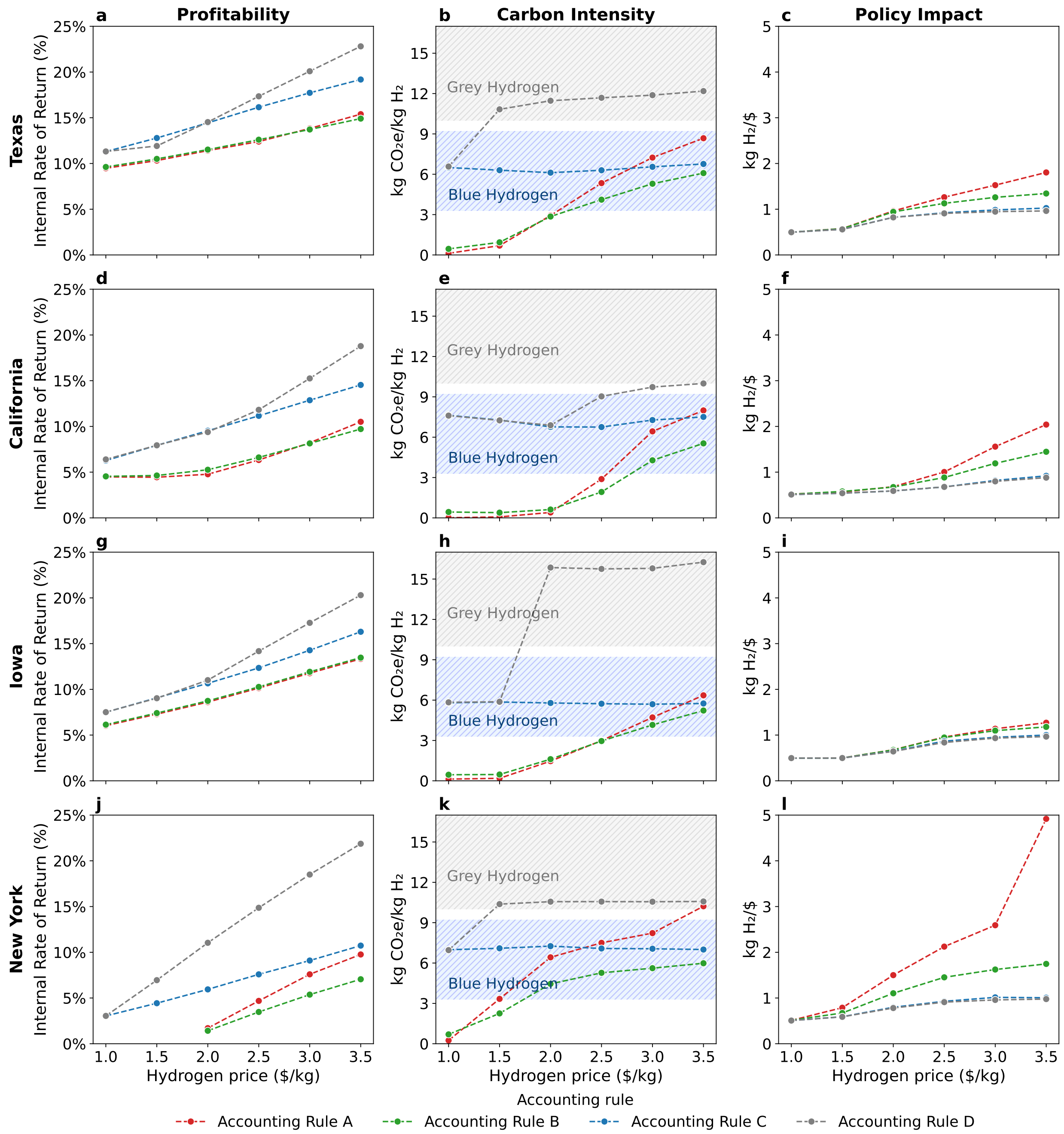

Supplementary Figure 10. Life-cycle performance in different states. This figure shows the impact calibrating the model to the current economic context of different states on (**a, d, g, j**) the profitability of PtG systems, (**b, e, h, k**) the life-cycle average carbon intensity of hydrogen, and (**c, f, i, l**) the policy impact of the production tax credit, given hydrogen prices between \$1.0 and \$3.5 per kg. Dots show the point estimates at specific hydrogen prices, while dashed lines interpolate between them for illustration. Results for Texas are shown for reference.

Supplementary Note 10. Alternative Degradation Rates

Alternative carbon accounting rules may affect the usage-based degradation of PtG systems differently. Since the techno-economic model relies on a time-based degradation of PtG systems of 1.0% per year[3;4], we estimate the magnitude of potential differences in usage-based degradation between carbon accounting rules by repeating the main analysis for annual degradation rates of 0.5% and 2.0%. As Supplementary Figure 11 shows, the profitability of PtG systems is slightly higher (lower) when the annual degradation rate is lower (higher), reflecting that the productive capacity becomes slightly more (less) productive. The life-cycle average carbon intensity of hydrogen and the policy impact of the tax credit remain almost unchanged.

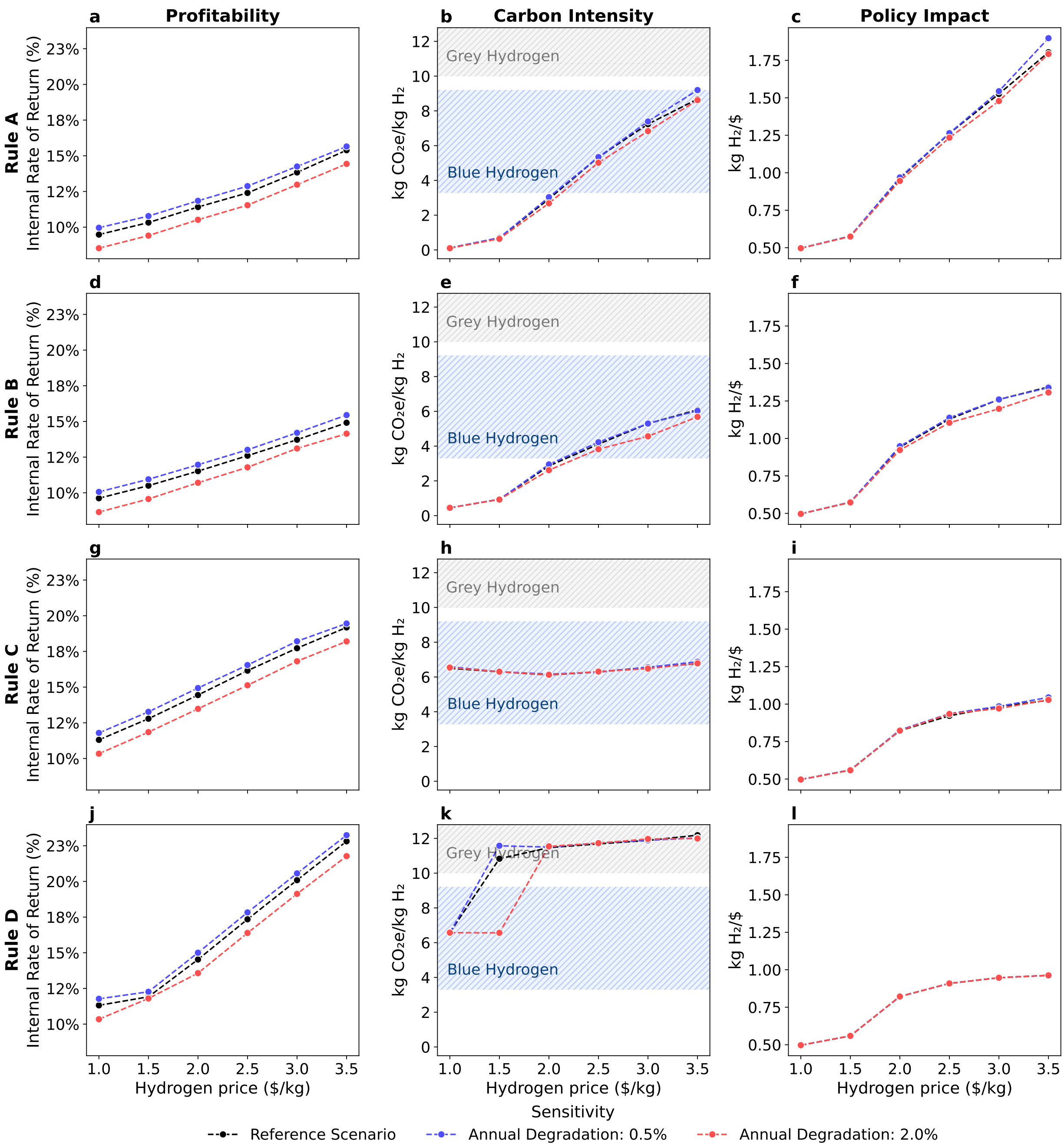

Supplementary Figure 11. Life-cycle performance under different degradation rates. This figure shows the impact of different degradation rates on the (**a, d, g, j**) profitability of PtG systems, (**b, e, h, k**) life-cycle average carbon intensity of hydrogen, and (**c, f, i, l**) policy impact of the production tax credit, given hydrogen prices between \$1.0 and \$3.5 per kg. Dots show the point estimates at specific hydrogen prices, while dashed lines interpolate between them for illustration. Our baseline specification of 1% per year is shown for reference.

Supplementary Note 11. Alkaline Electrolyzers

This section examines the possibility that PtG systems use an alkaline rather than a polymer electrolyte membrane (PEM) electrolyzer. We again run the analysis using the main specification described in the Methods of the main text. Changes in future prices and carbon intensity levels of general grid electricity would affect the results in direct analogy to the results in Supplementary Notes 6–8. We substitute the system price, fixed operating cost, and conversion rate. All other cost and operational parameters remain unchanged from the initial calibration. Supplementary Table 3 lists the changes in input parameters, with details provided in the Excel file available as part of the Supplementary Data.

Supplementary Table 3. Changes in input parameters.

in 2024 $US	**Unit**	**PEM**	**Alkaline**
System price, SP_h	$/kW	1,500.00	1,000.00
Fixed operating cost, F_{hi}	$/kW	45.00	25.00
Conversion rate, η	kg/kWh	0.0189	0.0185

Supplementary Figure 12 shows the resulting life-cycle performance of PtG systems with alkaline electrolyzers. Relative to PtG systems with PEM electrolyzers, we find that the profitability of PtG systems is now notably higher for all hydrogen prices and accounting rules considered. The life-cycle average carbon intensity of hydrogen and the policy impact of the tax credit are also notably higher for all hydrogen prices and accounting rules, except for the policy impact under accounting rule D. These increases mainly reflect that alkaline electrolyzers currently exhibit lower system prices than PEM electrolyzers. As a result, investors are incentivized to install larger electrolyzers and convert more general grid electricity. Overall, the relative impact of alternative carbon accounting rules remains largely unchanged.

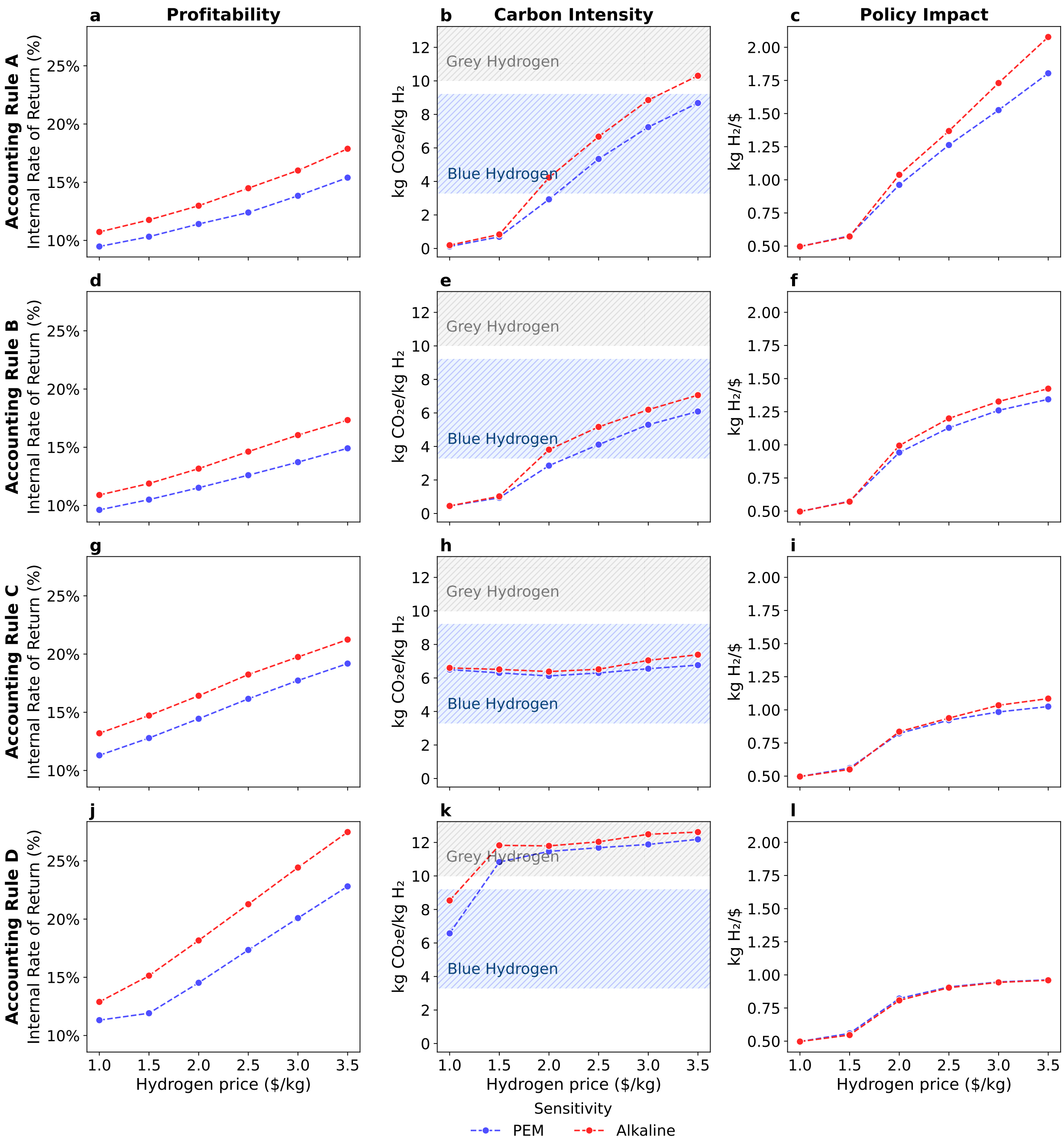

Supplementary Figure 12. Life-cycle performance of PtG systems with alkaline electrolyzers. If PtG systems were to use an alkaline rather than a PEM electrolyzer, this figure shows the resulting (**a, d, g, j**) profitability of PtG systems, (**b, e, h, k**) life-cycle average carbon intensity of hydrogen, and (**c, f, i, l**) policy impact of the production tax credit, given hydrogen prices between \$1.0 and \$3.5 per kg. Dots show the point estimates at specific hydrogen prices, while dashed lines interpolate between them for illustration. Results for PEM electrolyzers are shown for reference.

Supplementary Note 12. Alternative Electrolyzer Costs

This section examines the consequences of lower system prices for PtG units than assumed in the reference scenario. We again run the main specification described in the Methods of the main text, but alter the system price and fixed operating cost of the PtG unit. All other cost and operational parameters remain unchanged from the initial calibration. Supplementary Table 4 lists the changes in input parameters, with details provided in the Excel file available as part of the Supplementary Data.

Supplementary Table 4. Changes in cost parameters.

in 2024 $US	**Unit**	**PEM**	**Alkaline**
System price, SP_h (reference scenario)	$/kW	1,500.00	1,000.00
System price, SP_h (medium)	$/kW	1,000.00	500.00
System price, SP_h (low)	$/kW	500.00	200.00
Fixed operating cost, F_{hi} (reference scenario)	$/kW	45.00	25.00
Fixed operating cost, F_{hi} (medium)	$/kW	30.00	12.50
Fixed operating cost, F_{hi} (low)	$/kW	15.00	5.00

Supplementary Figures 13 and 14 show the resulting life-cycle performance of PtG systems with PEM and alkaline electrolyzers, respectively, at lower capacity-related costs. Relative to the reference scenario, we find that these cost reductions generally increase the profitability of PtG systems and, under accounting rules A-C, slightly increase the policy impact of the tax credit.

The cost reductions also lead to slightly higher life-cycle average carbon intensity levels for hydrogen under accounting rules A and B. This reflects that investors are incentivized to build larger PtG units and convert more grid electricity. Under accounting rules C and D, the life-cycle average carbon intensity levels for hydrogen remain mostly unchanged because the PtG systems already fully utilize capacity during most hours. Yet, when capacity-related costs of electrolyzers are sufficiently low or hydrogen prices sufficiently high, life-cycle average carbon intensity levels increase, as PtG systems are incentivized to forgo the tax credit in favor of higher conversion of general grid electricity. We observe this effect for PtG systems with PEM (alkaline) electrolyzers under accounting rule C at a hydrogen price above $2.5 per kg ($2.0 per kg).

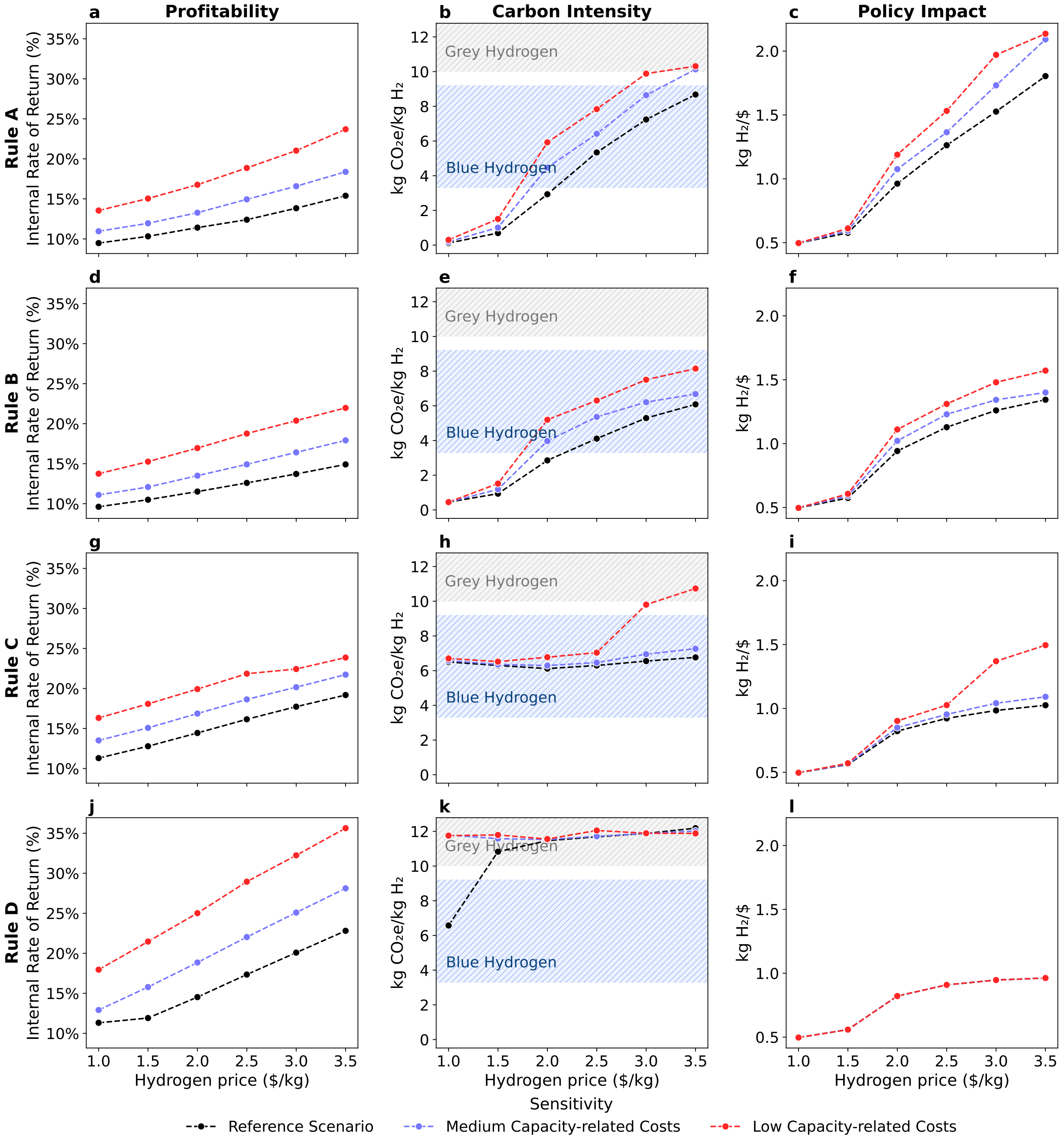

Supplementary Figure 13. Life-cycle performance of PtG systems with PEM electrolyzers at lower capacity-related costs. If PtG units were to have lower capacity-related costs, this figure shows the resulting (**a, d, g, j**) profitability of PtG systems, (**b, e, h, k**) life-cycle average carbon intensity of hydrogen, and (**c, f, i, l**) policy impact of the production tax credit, given hydrogen prices between \$1.0 and \$3.5 per kg. Dots show the point estimates at specific hydrogen prices, while dashed lines interpolate between them for illustration.

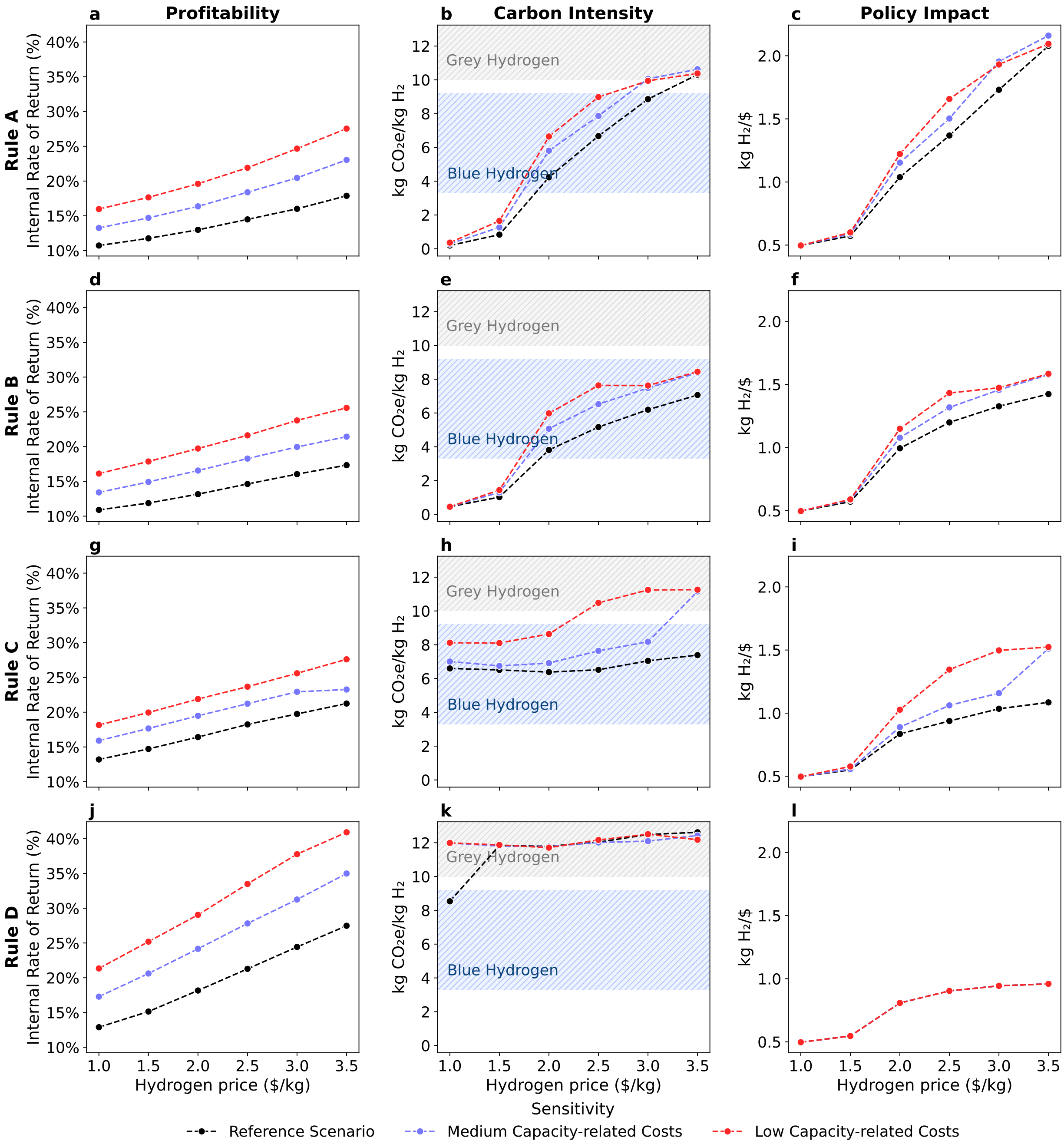

Supplementary Figure 14. Life-cycle performance of PtG systems with alkaline electrolyzers at lower capacity-related costs. If PtG units were to have lower capacity-related costs, this figure shows the resulting (**a, d, g, j**) profitability of PtG systems, (**b, e, h, k**) life-cycle average carbon intensity of hydrogen, and (**c, f, i, l**) policy impact of the production tax credit, given hydrogen prices between \$1.0 and \$3.5 per kg. Dots show the point estimates at specific hydrogen prices, while dashed lines interpolate between them for illustration.

Supplementary Note 13. Marginal Emission Rates

Consistent with regulatory mechanisms such as the US Inflation Reduction Act, the analysis has used average emission rates for general grid electricity. To assess the impact of marginal emission rates, we repeat the analysis for the main specification using the Cambium dataset from NREL[15;16]. Specifically, we replace the vector of carbon intensity levels for general grid electricity in the first inner optimization (i.e., the first year of operation onward) with short-run marginal emission rates from the Cambium mid-case scenario for the year 2025, and in the second inner optimization (i.e., the eleventh year of operation onward) with the corresponding rates for 2035. For consistency, we also replace the vectors of wholesale electricity prices in both inner optimizations with hourly marginal system costs (i.e., the parameter total_cost_busbar) from the Cambium mid-case scenario for 2025 and 2035.

Since the estimates in Cambium differ structurally from the observations used in the reference scenario, we repeat the preceding analysis using the respective average emission rates from Cambium. This allows us to gauge the impact of marginal emission rates only. We also provide the reference scenario for comparison.

Supplementary Figure 15 shows that the profitability of PtG systems and the policy impact of the tax credits based on either marginal or average emission rates from Cambium are almost identical. In contrast, the life-cycle average carbon intensity of hydrogen is significantly higher when marginal rather than average emission rates are used. This reflects that marginal emission rates are at least as high as average emission rates. Under carbon accounting rules A and B, the effect occurs especially for higher hydrogen prices, as PtG systems then convert more general grid electricity.

Relative to the reference scenario, profitability levels are generally lower under the scenarios based on Cambium. This mainly reflects that wholesale electricity prices are more frequently close to zero in the Cambium scenarios and thus the renewable energy sources achieve lower revenues. This effect is mitigated at higher hydrogen prices when electricity is converted to hydrogen more frequently. Similarly, the life-cycle average carbon intensity of hydrogen is generally lower under the scenarios based on average emission rates from Cambium. This is mainly due to the projected rapid decarbonization of the grid by 2035 in the Cambium mid-case scenario. Based on marginal emission rates from Cambium, the life-cycle average carbon intensity of hydrogen turns out to be fairly close to (notably higher than) the reference scenario under accounting rules A and B (C and D). The policy impact of the tax credit turns out to be close to the reference scenario in most variations considered.

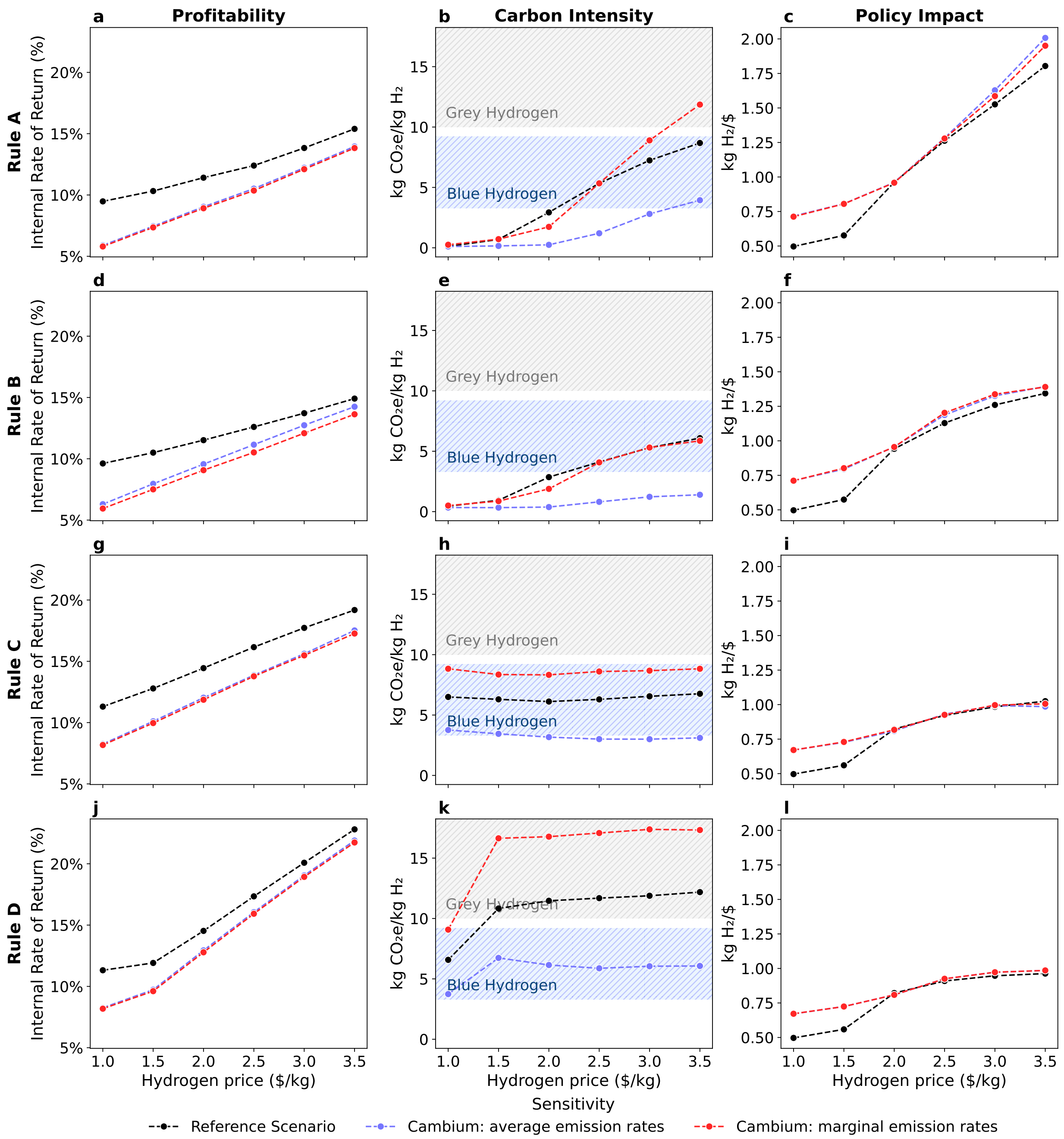

Supplementary Figure 15. Life-cycle performance under marginal emission rates. This figure shows the impact of using marginal emission rates for general grid electricity on the (**a, d, g, j**) profitability of PtG systems, (**b, e, h, k**) life-cycle average carbon intensity of hydrogen, and (**c, f, i, l**) policy impact of the production tax credit, given hydrogen prices between \$1.0 and \$3.5 per kg. Dots show the point estimates at specific hydrogen prices, while dashed lines interpolate between them for illustration. Results for PEM electrolyzers are shown for reference.

Supplementary References

[1] Michalski, J. *et al.* Hydrogen generation by electrolysis and storage in salt caverns: Potentials, economics and systems aspects with regard to the German energy transition. *International Journal of Hydrogen Energy* **42**, 13427–13443 (2017).

[2] Tax Foundation. State corporate income tax rates and brackets for 2020 (2020).

[3] DOE. DOE global energy storage database (2019).

[4] Buttler, A. & Spliethoff, H. Current status of water electrolysis for energy storage, grid balancing and sector coupling via power-to-gas and power-to-liquids: A review. *Renewable and Sustainable Energy Reviews* **82**, 2440–2454 (2018).

[5] Lazard. LCOE - Levelized Cost of Energy. Tech. Rep. (2024).

[6] LBNL. Land-Based Wind Market Report | Energy Markets & Policy. Tech. Rep., Lawrence Berkeley National Laboratory (2024).

[7] LBNL. Tracking the Sun | Energy Markets & Policy. Tech. Rep., Lawrence Berkeley National Laboratory (2024).

[8] LBNL. Utility-Scale Solar | Energy Markets & Policy. Tech. Rep., Lawrence Berkeley National Laboratory (2024).

[9] Hitachi Energy. Velocity suite - market intelligence services (2024).

[10] 117th United States Congress. H.R.5376 - inflation reduction act of 2022 (IRA): Public law no: 117-169 (2022).

[11] NREL *et al.* Updated Manufactured Cost Analysis for Proton Exchange Membrane Water Electrolyzers. Tech. Rep., National Renewable Energy Laboratory (2024).

[12] Glenk, G., Holler, P. & Reichelstein, S. Advances in power-to-gas technologies: Cost and conversion efficiency. *Energy & Environmental Science* **16**, 6058–6070 (2023).

[13] EIA. Opendata - U.S. Energy Information Administration (EIA). https://www.eia.gov/opendata/ (2024).

[14] Gagnon, P. *et al.* Standard Scenarios Report: A U.S. Electricity Sector Outlook. Tech. Rep., National Renewable Energy Laboratory (2024).

[15] NREL & Gagnon, Pieter; Pedro Andres Sanchez Perez; Julian Florez; James Morris; Marck Llerena Velasquez; And Jordan Eisenman. Cambium 2024 Data (2024).

[16] Gagnon, P. *et al.* Cambium 2024 Scenario Descriptions and Documentation. Technical Report, NREL (2025).
