# Supplementary material for: How carbon accounting rules shape incentives for hydrogen production
Source: Nat Commun. 2026 Aug 10;17:7260. doi: 10.1038/s41467-026-75473-z (PMC13457867; doi:10.1038/s41467-026-75473-z)
Supplement: Supplementary file 2 [file 41467_2026_75473_MOESM2_ESM.pdf]

Description of Additional Supplementary Files

File Name: Supplementary Data 1

Description: Contains model input data, processed results, and source data underlying the main and supplementary figures.
