# Supplementary material for: How carbon accounting rules shape incentives for hydrogen production
Source: Nat Commun. 2026 Aug 10;17:7260. doi: 10.1038/s41467-026-75473-z (PMC13457867; doi:10.1038/s41467-026-75473-z)
Supplement: Supplementary file 3 [file 41467_2026_75473_MOESM4_ESM.pdf]

nature portfolio

Peer Review File

How Carbon Accounting Rules Shape Incentives for Hydrogen Production

Corresponding Author: Professor Gunther Glenk

This file contains all editorial decision letters in order by version, followed by all author rebuttals in order by version.

Version 0:

Reviewer comments:

Reviewer #1

(Remarks to the Author)
General

This paper examines how alternative carbon accounting rules affect the financial and emission performance of Power-to-Gas (PtG) systems under policy support programs, specifically analyzing the US Inflation Reduction Act's production tax credit. While the research question is timely and relevant given the global policy focus on green hydrogen, the study suffers from significant methodological limitations that undermine the validity and generalizability of its findings.
The paper's investor perspective represents a valuable contribution to the literature, which has predominantly focused on system-wide optimization approaches. However, the reliance on historical data to project future performance over 20-30 year investment horizons, combined with geographic limitations and oversimplified assumptions, severely constrains the practical relevance of the results.

Major Concerns
1. Temporal Validity and Data Limitations
The most significant flaw in this study is the use of historical electricity prices and carbon intensity data (2019-2023) to evaluate hydrogen production performance over 20-30 year investment periods. This approach is particularly problematic because the highest carbon intensity results appear 10-15 years after the tax credit expires, when the electricity grid will likely be substantially decarbonized. The sensitivity analysis in the supplementary material inadequately addresses this issue by varying electricity prices and carbon intensity separately, when these parameters are inherently coupled—higher renewable penetration typically results in both lower carbon intensity and lower electricity prices simultaneously. Future scenarios should reflect realistic projections based on policy targets or established energy transition studies rather than historical variations.
2. Geographic Limitations and Generalizability
The analysis focuses exclusively on Texas, using a single set of wind and solar capacity factors, yet the text suggests broader applicability to the entire United States and even internationally. This is misleading given the substantial variation in grid carbon intensity, renewable resources, and electricity market structures across different states and regions. Previous studies have clearly demonstrated the critical dependence of results on background grid characteristics. The paper should either expand the geographic scope or clearly limit its conclusions to the specific system studied.
3. System Boundary and Grid Interaction Issues
The study adopts a narrow investor perspective that ignores critical interactions with the background electricity grid. With large-scale electrolytic hydrogen production, dedicated renewable generation might simply displace other renewable sources in the general grid rather than provide additional decarbonization benefits. Furthermore, increased electricity demand from electrolysis could trigger higher utilization of conventional power plants, increasing overall grid carbon intensity. These system-wide effects are particularly relevant for policymakers who need to understand net emission impacts rather than accounting-based metrics for individual plants.
4. Temporal aggregation
The temporal aggregation to 1000 randomly selected time steps represents an overly simplification without proper validation. More sophisticated time series aggregation methods, such as those available for example in the tsam Python package, should be employed to preserve important temporal patterns. Additionally, robustness testing with higher temporal resolution is essential to validate the results.

Additional Issues
1. Clarity and Scope Definition
The paper should more clearly state that findings apply specifically to the US system and cannot be easily transferred to other regions with different grid characteristics. Statements such as "Contrary to common beliefs, we find that more stringent accounting rules provide investors with sufficient incentives to invest in PtG systems today" should be qualified to avoid overstating the geographic applicability of the results.
2. Missing Technical Details in the pdf
Critical technology assumptions including investment costs, fixed and variable operation and maintenance costs, electrolysis efficiency, renewable capacity factors, and electricity price assumptions should be included as a parameter table in the supplementary material pdf so that one does not have to download an excel file and search for the most important assumptions.
3. Literature Gaps
The paper omits important recent literature on hydrogen policy analysis and grid interaction effects, including relevant work who have addressed similar questions, e.g.
- Brauer J, Villavicencio M and Trüby J 2022 Green hydrogen: How grey can it be? Technical Report (EUI Florence School of Regulation)
- Ruhnau O and Schiele J 2022 Flexible green hydrogen: Economic benefits without increasing power sector emissions Technical Report (ZBW, Leibniz Information Centre for Economics) Older version: http://hdl.handle.net/10419/253267
- Zeyen E, Riepin I and Brown T 2024 Temporal regulation of renewable supply for electrolytic hydrogen
4. Issues in Excel file
In Supplemntary Data Excel sheet Outputs some headlines seem to be wrong (e.g. line 19 should probably be accounting Rule A/B/C/D not A/B/A/B)
5. Figure caption
Methodology for calculating life-cycle average carbon intensity in Figure 2b lacks clear explanation. Here again it really should be considered that background grid is going to decarbonise further in the next years and carbon intensity is going to reduce, especially because the larger carbon intensity is after the period of the tax credit which is in more than 10 years time, again taking historical values from 2019-2023 is not going to give meaningful results.
6. Explanation of some results
Dedicated renewable generation seems to be mainly relying on wind, what is the reason and the impact of the accounting rules?

(Remarks on code availability)
I only checked if the Github repository is available and one can pull it. I haven't checked the code.

Reviewer #2

(Remarks to the Author)
There are lots of usefule work and interesting conclusions in the paper ,however, I think the conclusions may differ with more range of detail input datas:
1. Need more details especially performance data for PEM electrolyzers, i.e, I-V curve, allowable load range with safety garantee, power control strategy(constant power or constant current).
2. if PtG with ALk, what the conclusions will be like?
3. Does grid company policy or rules have effect on the conclusions?
4.According to the results analysis, different hydrogen price lead to different effect of accounting rules, which is obivious with high hydrogen price, but seems little difference with hydrogen price lower than 1.5$/kg， however the hydrogen cost target is 1$/kg by 2030 with US DOE.

(Remarks on code availability)

Reviewer #3

(Remarks to the Author)
1.While the topic addressed in this manuscript is highly relevant and the research question is timely, the novelty could be better positioned relative to existing literature on hydrogen carbon accounting and PtG investment economics. The authors should explicitly acknowledge related studies and clarify what unique insight their work contributes beyond the current state of knowledge. A more detailed comparison against key references would help strengthen the claim of originality and significance.

2.The conclusions drawn about policy effectiveness are interesting, but they rely heavily on a simplified electricity system representation that may not adequately reflect real-world operational constraints or marginal emission dynamics. At present, it is unclear whether the findings would still hold under varying grid decarbonization trajectories, locational factors, or alternative electricity sourcing strategies. Expanding the scenario space or incorporating uncertainty analysis is needed to justify the robustness of the claims.

3.The methodology relies on an economic model that maximizes investor net present value, but important details of the optimization model are missing or insufficiently described to ensure full reproducibility. I recommend adding clearer mathematical formulations, variable definitions, solver specifications, and sensitivity calibration procedures. Transparency in these aspects is essential for reviewers and readers to evaluate the rigor of the modeling framework.

4.The assumptions underlying the carbon intensity calculations should be more rigorously justified and documented. For instance, the exclusion of infrastructure manufacturing emissions and the reliance on average grid emission factors may materially bias results. Including sensitivity analysis or alternative accounting boundary scenarios would increase the credibility of the environmental assessment and help readers understand the limits of applicability.

5.The manuscript currently provides few checks that the model-based outputs align with empirical data or other authoritative benchmarks. To ensure that results are meaningful to policy decisions, the authors should conduct validation exercises, such as comparing profitability outcomes to existing PtG pilot projects or benchmarking carbon intensity values against certified hydrogen pathways. Without such validation, it is difficult to assess the reliability of the results.

6.The presentation of findings is mostly narrative and would benefit strongly from more comprehensive figures and tables summarizing model behavior, scenario differences, and investment–emissions trade-offs. Clear visualization of critical results (e.g., IRR vs. CI trade-offs, cost breakdowns, and sensitivity responses) would help editors and readers appreciate the practical implications and the decision value of the work.

7.Finally, the policy implications section should be significantly strengthened. Although the manuscript highlights potential risks of current carbon accounting rules, it falls short of providing actionable policy recommendations. It would improve the impact of the paper if the authors could propose specific regulatory mechanisms, enforcement strategies, or safeguards to prevent unintended consequences such as increased fossil-based electricity use. Policy relevance is essential for a journal publishing in this space.

(Remarks on code availability)

Version 1:

Reviewer comments:

Reviewer #2

(Remarks to the Author)
1. The cost of PEM and ALK used in this paper are 1500$/KW and 1000$/kW respectively, which are a few times higher than the market price(I think 500 and 200 are globally reasonable), which may lead different conclusions .
2 W/O energy storage are very important to P2G system and hydrogen production cost, and there are lots scenarios for Power to hydrogen(such as W/O energy storage, off/on grid, electrolysers allowable working load range), which should be investigated carefully,and may cause hydrogen production cost varies much.
Generally speaking , according to the current calculations and results, it's hard to say the conclusions are universal.

(Remarks on code availability)

Reviewer #3

(Remarks to the Author)
Comments for Author
Thank you for the revised manuscript and the detailed response.
This revised manuscript addresses a timely and highly policy-relevant question: how alternative carbon accounting rules for electrolytic hydrogen shape investor behavior and the resulting divergence between certified and actual emissions outcomes. The investor-centric, nested optimization framework is a valuable complement to system-planning studies, because it explicitly captures private incentives and strategic responses to accounting constraints.
That said, several remaining assumptions substantially affect the credibility and generality of the quantitative results and, in particular, the policy interpretation. I therefore recommend major revision.
Major comments (major revision)
1. Extrapolation from historical hourly data
The analysis extrapolates long-run outcomes from historical hourly price and emissions patterns. In high-renewables futures, hourly dynamics are likely to differ qualitatively (e.g., more frequent negative prices, higher volatility, altered price–emissions correlations), which could materially change optimal operation and emissions outcomes.
The authors should either demonstrate robustness using at least one forward-looking or stylized hourly scenario that alters the joint distribution of prices and emissions, or explicitly limit quantitative conclusions to the historical-hourly setting.
2. Emissions attribution: average versus marginal effects
Key conclusions about “actual” emissions hinge on the choice of emissions factor. When additional electricity demand affects dispatch, average emissions may diverge substantially from marginal impacts.
The manuscript would benefit from clearer justification of the chosen accounting approach and from a sensitivity analysis using marginal emissions (or a defensible proxy), with discussion of implications for the certified–actual divergence..
3. Exogenous grid assumption
Electricity prices, dispatch, and emissions are treated as exogenous. At scale, PtG deployment may affect all three, which is central to assessing policy effectiveness and actual emissions outcomes.
Either a simplified endogenous grid-response representation should be introduced, or the policy conclusions should be

more tightly bounded to settings in which grid feedbacks are negligible.
4. Cross-regional generalizability
Claims about generalizability across states depend critically on renewable resource profiles and their covariance with prices and emissions. Varying prices and emissions while holding capacity factors fixed risks biasing profitability and emissions comparisons.
This limitation should be addressed using state-specific capacity-factor time series where possible, or more clearly acknowledged in the interpretation of cross-state results.
5. Credit-forgoing behavior at high hydrogen prices
The manuscript highlights that investors may rationally forgo tax credits and rely on grid electricity at high hydrogen prices. This mechanism is potentially important for policy design but is currently described only qualitatively.
Quantifying the conditions under which this behavior emerges would strengthen the argument.
6. Operational realism: cycling and degradation
Different accounting rules imply different utilization and cycling patterns, which may affect electrolyzer degradation and replacement. Ignoring these effects could bias IRR comparisons across rules.
At least one sensitivity case incorporating simplified degradation or cycling costs would help assess the robustness of the main conclusions.
7. Interpretation of policy metrics
The “Policy Impact” metric measures hydrogen output per unit subsidy and may appear favorable under relaxed rules even when emissions are high. This risks being misinterpreted as an environmental performance indicator.
The manuscript should clarify the interpretation and limits of this metric and, ideally, complement it with an emissions-normalized measure (e.g., subsidy per unit of emissions abated).
8.Minor comments
Please double-check consistency of variable names, units, and definitions across the main text and Supplementary.
Where time-sensitive regulatory timelines are mentioned, please ensure citations are provided and wording is appropriately bounded.
Recommendation
Major revision. The paper addresses an important question and offers a valuable modeling perspective. However, the quantitative results and policy interpretation require stronger robustness analysis and/or more explicit bounding of claims, particularly regarding future hourly dynamics, emissions attribution, and grid feedbacks.

(Remarks on code availability)

Version 2:

Reviewer comments:

Reviewer #3

(Remarks to the Author)
The overall structure of the manuscript is clear, the methodology is rigorous, and the core conclusions are consistent with the modeling framework. I recommend a minor revision to further improve the quality of the paper：
Figure 1 is helpful conceptually, but its visual design could be improved for better clarity and aesthetic quality.

(Remarks on code availability)

Version 3:

Reviewer comments:

Reviewer #3

(Remarks to the Author)
No further comments. Overall, the manuscript is well structured and the presentation is clear.

(Remarks on code availability)
I have briefly checked the provided GitHub repository but have not independently run the code. Therefore, I cannot fully assess the reproducibility of the results based on the code.

Open Access This Peer Review File is licensed under a Creative Commons Attribution 4.0 International License, which permits use, sharing, adaptation, distribution and reproduction in any medium or format, as long as you give appropriate credit to the original author(s) and the source, provide a link to the Creative Commons license, and indicate if changes were made.
In cases where reviewers are anonymous, credit should be given to 'Anonymous Referee' and the source.
The images or other third party material in this Peer Review File are included in the article's Creative Commons license, unless indicated otherwise in a credit line to the material. If material is not included in the article's Creative Commons license and your intended use is not permitted by statutory regulation or exceeds the permitted use, you will need to obtain permission directly from the copyright holder.
To view a copy of this license, visit https://creativecommons.org/licenses/by/4.0/

Authors' Response to Reviewers on
"Policy Support for Electrolytic Hydrogen: Impact of Alternative Carbon Accounting Rules"
Ms. Ref. No.: NCOMMS-25-55376-T, *Nature Communications*

We are grateful to the reviewers for their many constructive comments and suggestions. In response, we have conducted a comprehensive revision of our manuscript. In particular, we now include additional geographic regions in our analysis and examine more comprehensively the impact of alternative future electricity prices and emission intensity levels of grid electricity. We have also clarified our contributions in relation to recent literature additions, further explaining specific aspects of our methodology and results, and providing actionable policy recommendations. We are confident that the revision has resulted in a stronger and more transparent paper.

The following points respond to each reviewer's comments in detail. For that purpose, the original reviewer comments have been italicized, while our responses are shown in regular font.

Response to Reviewer 1

# General

This paper examines how alternative carbon accounting rules affect the financial and emission performance of Power-to-Gas (PtG) systems under policy support programs, specifically analyzing the US Inflation Reduction Act's production tax credit. While the research question is timely and relevant given the global policy focus on green hydrogen, the study suffers from significant methodological limitations that undermine the validity and generalizability of its findings.

The paper's investor perspective represents a valuable contribution to the literature, which has predominantly focused on system-wide optimization approaches. However, the reliance on historical data to project future performance over 20-30 year investment horizons, combined with geographic limitations and oversimplified assumptions, severely constrains the practical relevance of the results.

# Major Concerns

1. Temporal Validity and Data Limitations:

The most significant flaw in this study is the use of historical electricity prices and carbon intensity data (2019-2023) to evaluate hydrogen production performance over 20-30 year investment periods. This approach is particularly problematic because the highest carbon intensity results appear 10-15 years after the tax credit expires, when the electricity grid will likely be substantially decarbonized. The sensitivity analysis in the supplementary material inadequately addresses this issue by varying electricity prices and carbon intensity separately, when these parameters are inherently coupled—higher renewable penetration typically results in both lower carbon intensity and lower electricity prices simultaneously. Future scenarios should reflect realistic projections based on policy targets or established energy transition studies rather than historical variations.

We agree that future electricity prices and the emission intensity levels of grid electricity are likely to change simultaneously. To examine the impact of these dynamics, we have added new scenarios

that incorporate projections of future grid electricity prices and emission intensity levels corresponding to alternative energy transition pathways.

Since the exact energy transition pathway remains uncertain, and our analysis focuses on the impact of alternative carbon accounting rules, we have kept our analysis in the main body based on historical grid electricity prices and emission intensity levels. Yet, the main body discusses the changes to our main findings resulting from alternative future electricity price dynamics and emission intensity levels of grid electricity. Details on the findings from the new scenarios are provided in Supplementary Notes 5-7.

2. Geographic Limitations and Generalizability
The analysis focuses exclusively on Texas, using a single set of wind and solar capacity factors, yet the text suggests broader applicability to the entire United States and even internationally. This is misleading given the substantial variation in grid carbon intensity, renewable resources, and electricity market structures across different states and regions. Previous studies have clearly demonstrated the critical dependence of results on background grid characteristics. The paper should either expand the geographic scope or clearly limit its conclusions to the specific system studied.

We also agree that differences in the technological and economic environments of different states and regions could substantially change our results. To examine this possibility, we have rerun our analysis for California, Iowa, and New York. These states, and the corresponding electricity grid systems, differ considerably in terms of their current power generation mix, wholesale market prices and carbon intensity levels of general grid electricity, and availability of wind and solar resources.

Our expanded results suggest that the simultaneous changes in input parameters associated with each state change the absolute profitability and carbon intensity estimates of electrolytic hydrogen production. Yet, our main findings about the relative impact of alternative carbon accounting rules are consistent across states. This reflects that our analysis relies on an embedded optimization algorithm that determines the cost-efficient configuration and utilization of renewable energy and hydrogen production capacity.

The revised manuscript reports our main findings based on our initial calibration in the context of Texas. Yet, we now discuss the extent to which our findings generalize to other states in the Discussion section. Details on the new scenarios are provided in Supplementary Note 8.

3. System Boundary and Grid Interaction Issues
The study adopts a narrow investor perspective that ignores critical interactions with the background electricity grid. With large-scale electrolytic hydrogen production, dedicated renewable generation might simply displace other renewable sources in the general grid rather than provide additional decarbonization benefits. Furthermore, increased electricity demand from electrolysis could trigger higher utilization of conventional power plants, increasing overall grid carbon intensity. These

system-wide effects are particularly relevant for policymakers who need to understand net emission impacts rather than accounting-based metrics for individual plants.

We further agree that electricity demand from electrolyzers could absorb a large share of existing renewable power generation and also lead to higher emissions from conventional power plants. The first concern is precisely the reason why our analysis initially (i.e., for accounting rules A-C) requires the renewable energy capacity to be incremental, yet this requirement is relaxed for accounting rule D. The second concern reflects a primary motivation for our study. Central to our analysis is the examination of how alternative carbon accounting rules shape incentives for investors to utilize electrolyzer capacity by procuring additional, carbon-intensive electricity from the grid.

We acknowledge that our analysis considers the installed capacity and hourly generation of alternative power units in the grid as exogenous. As we note in the Conclusion, an important avenue for future research is to develop a (partial) market equilibrium model that endogenizes these decisions and allows for the analysis of how alternative carbon accounting rules affect total emissions from electricity and hydrogen production.

4. Temporal aggregation

The temporal aggregation to 1000 randomly selected time steps represents an overly simplification without proper validation. More sophisticated time series aggregation methods, such as those available for example in the tsam Python package, should be employed to preserve important temporal patterns. Additionally, robustness testing with higher temporal resolution is essential to validate the results.

We note that all our main specifications are based on the full annual dataset of 8760 hours. We only used a subset of 1000 randomly selected hours for supplementary calculations to substantially reduce computing time. We had decided to use this subset after tests had shown that the differences between output variables based on 8760 hours and 1000 hours are small. We elaborate on these tests in Supplementary Note 2.

# Additional Issues

1. Clarity and Scope Definition

The paper should more clearly state that findings apply specifically to the US system and cannot be easily transferred to other regions with different grid characteristics. Statements such as "Contrary to common beliefs, we find that more stringent accounting rules provide investors with sufficient incentives to invest in PtG systems today" should be qualified to avoid overstating the geographic applicability of the results.

We have revised the exposition in the paper to distinguish findings specific to the regions we analyzed from conclusions that may be generalized more broadly.

2. Missing Technical Details in the pdf

Critical technology assumptions including investment costs, fixed and variable operation and maintenance costs, electrolysis efficiency, renewable capacity factors, and electricity price assumptions should be included as a parameter table in the supplementary material pdf so that one does not have to download an excel file and search for the most important assumptions.

As suggested, we now provide such information in Supplementary Note 1.

3. *Literature Gaps*

The paper omits important recent literature on hydrogen policy analysis and grid interaction effects, including relevant work who have addressed similar questions, e.g.

- Brauer J, Villavicencio M and Trüby J 2022 Green hydrogen: How grey can it be? Technical Report (EUI Florence School of Regulation)

- Ruhnau O and Schiele J 2022 Flexible green hydrogen: Economic benefits without increasing power sector emissions Technical Report (ZBW, Leibniz Information Centre for Economics) Older version: http://hdl.handle.net/10419/253267

- Zeyen E, Riepin I and Brown T 2024 Temporal regulation of renewable supply for electrolytic hydrogen

We thank the reviewer for these references and included them in the revised paper.

4. *Issues in Excel file*

In Supplemntary Data Excel sheet Outputs some headlines seem to be wrong (e.g. line 19 should probably be accounting Rule A/B/C/D not A/B/A/B)

Thank you for bringing this to our attention. We corrected the mislabeled headings.

5. *Figure caption*

Methodology for calculating life-cycle average carbon intensity in Figure 2b lacks clear explanation. Here again it really should be considered that background grid is going to decarbonise further in the next years and carbon intensity is going to reduce, especially because the larger carbon intensity is after the period of the tax credit which is in more than 10 years time, again taking historical values from 2019-2023 is not going to give meaningful results.

The life-cycle carbon intensity in Figure 2b is calculated based on equation [1]. We have included a corresponding note in the caption to the figure.

6. *Explanation of some results:*

Dedicated renewable generation seems to be mainly relying on wind, what is the reason and the impact of the accounting rules?

The primary reason for this is that, in the examined geographic region, wind energy offers higher and more stable generation per unit of installed capacity compared to solar energy. This enables

more zero-emission hydrogen production and higher earnings from production tax credits. We hope this feature becomes clearer in the revised paper.

Response to Reviewer 2

There are lots of usefule work and interesting conclusions in the paper ,however, I think the conclusions may differ with more range of detail input datas:

1. *Need more details especially performance data for PEM electrolyzers, i.e, I-V curve, allowable load range with safety garantee, power control strategy(constant power or constant current).*

As suggested, the revised manuscript provides more details on the operational performance of PEM electrolyzers in the subsection Model Calibration in Methods.

2. *if PtG with ALk, what the conclusions will be like?*

We have rerun our analysis based on alkaline electrolyzers. Our calculations show that the resulting profitability of PtG systems and carbon intensity levels of the hydrogen produced are notably higher than those for PEM electrolyzers. This mainly reflects that alkaline electrolyzers currently exhibit lower system prices than PEM electrolyzers. As a result, investors are incentivized to install larger electrolyzers and convert more general grid electricity. A summary of this analysis is provided in Supplementary Note 9. Details can be generated using our publicly accessible computational code.

3. *Does grid company policy or rules have effect on the conclusions?*

Grid company policies and rules may indeed affect our results. To examine this possibility, we have rerun our analysis for California, Iowa, and New York. These states, and the corresponding electricity grid systems, differ considerably in terms of their current power generation mix, wholesale market prices and carbon intensity levels of general grid electricity, and availability of wind and solar resources.

Our expanded results suggest that the simultaneous changes in input parameters associated with each state change the absolute profitability and carbon intensity estimates of electrolytic hydrogen production. Yet, our main findings about the relative impact of alternative carbon accounting rules are consistent across states. This reflects that our analysis relies on an embedded optimization algorithm that determines the cost-efficient configuration and utilization of renewable energy and hydrogen production capacity.

The revised manuscript reports our main findings based on our initial calibration to Texas. Yet, we now briefly discuss the extent to which our findings generalize to other states in the Discussion. Details on the new scenarios are provided in Supplementary Note 8.

4. *According to the results analysis, different hydrogen price lead to different effect of accounting rules, which is obivious with high hydrogen price, but seems little difference with hydrogen price lower than 1.5$/kg， however the hydrogen cost target is 1$/kg by 2030 with US DOE.*

Under the different carbon accounting rules, the resulting operational and investment patterns vary considerably, especially at higher hydrogen prices but also at lower ones. While we agree that the $1/kg DOE target provides an important benchmark, current market prices for electrolytic hydrogen remain considerably higher (Terlouw et al., 2022; ICCT, 2024).

Response to Reviewer 3

1. *While the topic addressed in this manuscript is highly relevant and the research question is timely, the novelty could be better positioned relative to existing literature on hydrogen carbon accounting and PtG investment economics. The authors should explicitly acknowledge related studies and clarify what unique insight their work contributes beyond the current state of knowledge. A more detailed comparison against key references would help strengthen the claim of originality and significance.*

As suggested, we expanded the comparison to previous literature and clarified the contribution of our analysis to earlier work.

2. *The conclusions drawn about policy effectiveness are interesting, but they rely heavily on a simplified electricity system representation that may not adequately reflect real-world operational constraints or marginal emission dynamics. At present, it is unclear whether the findings would still hold under varying grid decarbonization trajectories, locational factors, or alternative electricity sourcing strategies. Expanding the scenario space or incorporating uncertainty analysis is needed to justify the robustness of the claims.*

We note that our analysis examines the impact of changes in future grid electricity prices and future emission intensity levels of grid electricity separately. To further examine the sensitivity of our results, we have also examined the impact of simultaneous changes in future prices and the emission intensity levels of grid electricity. We have also rerun our analysis for other major electricity grid systems in the US to explore the potential impact of locational differences.

Our revised results suggest that the changes in input parameters associated with the different scenarios change the absolute profitability and carbon intensity estimates of electrolytic hydrogen production. Yet, our main findings about the relative impact of alternative carbon accounting rules are quite robust. This robustness reflects that our analysis relies on an embedded optimization algorithm that determines the cost-efficient configuration and utilization of renewable energy and hydrogen production capacity. We discuss the results of these additional sensitivity tests in the Discussion in the main body of the manuscript and provide details in Supplementary Notes 5-8.

3. *The methodology relies on an economic model that maximizes investor net present value, but important details of the optimization model are missing or insufficiently described to ensure full reproducibility. I recommend adding clearer mathematical formulations, variable definitions, solver specifications, and sensitivity calibration procedures. Transparency in these aspects is essential for reviewers and readers to evaluate the rigor of the modeling framework.*

The analytical model underlying our analysis and its numerical calibration in Python are comprehensively described in Methods. The computational code is annotated, publicly accessible, interoperable, and reusable. All input parameters are provided in a Supplementary Excel file. As such, we hope our analysis is now fully transparent and reproducible.

4. *The assumptions underlying the carbon intensity calculations should be more rigorously justified and documented. For instance, the exclusion of infrastructure manufacturing emissions and the reliance on average grid emission factors may materially bias results. Including sensitivity analysis or alternative accounting boundary scenarios would increase the credibility of the environmental assessment and help readers understand the limits of applicability.*

As we note throughout the paper, our analysis focuses on carbon accounting rules that are widely discussed and described in recent guidance from regulators in the United States and the European Union. While we agree that it would be interesting to explore other carbon accounting rules, as stated in the Conclusion, it appears unlikely to us that regulators will adopt rules in the near future that will expand the responsibility of firms to indirect emissions beyond those embodied in the energy they consume.

5. *The manuscript currently provides few checks that the model-based outputs align with empirical data or other authoritative benchmarks. To ensure that results are meaningful to policy decisions, the authors should conduct validation exercises, such as comparing profitability outcomes to existing PtG pilot projects or benchmarking carbon intensity values against certified hydrogen pathways. Without such validation, it is difficult to assess the reliability of the results.*

We wish we could compare our results with existing pilot projects, yet at this stage, the requisite information on such projects is unavailable. We also note that the central element of our analysis is to assess how the "certified" carbon intensity of electrolytic hydrogen, as defined by regulators, compares with its "actual" carbon intensity. As such, the results of our study should be meaningful to policy decisions.

6. *The presentation of findings is mostly narrative and would benefit strongly from more comprehensive figures and tables summarizing model behavior, scenario differences, and investment–emissions trade-offs. Clear visualization of critical results (e.g., IRR vs. CI trade-offs, cost breakdowns, and sensitivity responses) would help editors and readers appreciate the practical implications and the decision value of the work.*

Figures 2 and 4 show the trade-off between the profitability (IRR) and carbon intensity of electrolytic hydrogen production across carbon accounting rules and hydrogen selling prices. Figures 3 and 5 further show the trade-off between the annual production and carbon intensity of electrolytic hydrogen across carbon accounting rules. Figure 6 shows the impact of alternative carbon accounting rules on the capacity of the policy support to incentivize electrolytic hydrogen production. In addition to these figures, we have added tables in the Supplementary Information that provide the main input parameters and output variables. Together, we believe these figures and tables comprehensively summarize and visualize our results.

7. *Finally, the policy implications section should be significantly strengthened. Although the manuscript highlights potential risks of current carbon accounting rules, it falls short of providing actionable policy recommendations. It would improve the impact of the paper if the authors could propose specific regulatory mechanisms, enforcement strategies, or safeguards to prevent unintended consequences such as increased fossil-based electricity use. Policy relevance is essential for a journal publishing in this space.*

We emphasize that our entire analysis is intended to be of immediate policy relevance as it examines the implications of alternative policy support programs for hydrogen. The Abstract, Introduction, and Conclusion synthesize actionable policy recommendations emerging from our analysis. The revised version has further clarified these throughout the manuscript.

In conclusion, we again thank the three reviewers for their many insightful comments and suggestions. We believe that the comprehensive revision has resulted in a more comprehensive and transparent manuscript with a sharper and more generalizable message. We hope you agree.

Authors' Response to Reviewers on
"Policy Support for Electrolytic Hydrogen: Impact of Alternative Carbon Accounting Rules"
Ms. Ref. No.: NCOMMS-25-55376-T, *Nature Communications*

We are grateful to the reviewers for their many constructive comments and suggestions. In response, we have substantially expanded our sensitivity and robustness tests. In particular, we have examined alternative electrolyzer costs and degradation rates, a forward-looking scenario for future market prices and grid carbon intensity levels, and the impact of marginal emission rates for general grid electricity. In addition, we have clarified the hourly operation of PtG systems under different carbon accounting rules. We have also carefully reviewed the manuscript to ensure that our conclusions are limited to the settings we examine.

As part of the revision, we have also addressed the editorial comments. In particular, we have changed the title to "Impact of Alternative Carbon Accounting Rules on Hydrogen Production" and removed the section keywords. We have also carefully reviewed the manuscript to ensure it complies with the journal guidelines. We are confident that these revisions have strengthened the robustness and transparency of the paper.

The following points respond to each reviewer's comments in detail. For that purpose, the original reviewer comments have been italicized, while our responses are shown in regular font.

Response to Reviewer 2

1. The cost of PEM and ALK used in this paper are 1500$/KW and 1000$/kW respectively, which are a few times higher than the market price(I think 500 and 200 are globally reasonable), which may lead different conclusions .

To examine the possibility of lower system prices for PtG units, we repeated our analysis using the main specification for two alternative scenarios where system prices are (i) $1000/kW for PEM and $500/kW for alkaline electrolyzers or (ii) $1000/kW for PEM and $200/kW for alkaline electrolyzers. Our calculations, reported in Supplementary Note 12, show that for most carbon accounting rules, the profitability of PtG systems, the life-cycle average carbon intensity of hydrogen produced, and the policy impact are now higher than in our reference scenario. This mainly reflects that PtG units have larger capacity sizes and convert more grid electricity. We find that the relative impact of the different carbon accounting rules remains unchanged across all variations considered.

2. W/O energy storage are very important to P2G system and hydrogen production cost, and there are lots scenarios for Power to hydrogen(such as W/O energy storage, off/on grid, electrolysers allowable working load range), which should be investigated carefully,and may cause hydrogen production cost varies much.

Generally speaking , according to the current calculations and results, it's hard to say the conclusions are universal.

We agree that our techno-economic analysis could be extended in several directions. For example, investors might install an energy storage unit. Provided that such an addition would be financially attractive, it would likely improve the life-cycle average carbon intensity of hydrogen produced and the policy impact of the tax credit. This would be the consequence of more renewable energy being converted to hydrogen or sold to the grid at higher prices.

Alternatively, the PtG system considered in our analysis could be disconnected from the general grid. Any hydrogen produced would then have a carbon intensity of zero. The profitability of the system would also be lower than in our baseline setting, because any excess renewable energy could not be sold to the grid, while any excess capacity of the PtG unit could not be utilized with electricity procured from the grid.

Finally, electrolyzers could be constrained in their load range. Such a constraint would likely reduce the profitability of PtG systems and the policy impact of the tax credit, mainly because less hydrogen would be produced.

We now discuss these extensions in the Discussion. We also emphasize that our analysis focuses on how alternative carbon accounting rules for assessing the carbon intensity of electrolytic hydrogen shape the financial and emission performance of PtG systems. Our robustness analysis suggests that variations in the basic input parameters alter the absolute life-cycle performance of PtG systems. Yet, such variations do not alter the relative impact of alternative carbon accounting rules, which are consistent across the different scenarios considered.

Response to Reviewer 3

This revised manuscript addresses a timely and highly policy-relevant question: how alternative carbon accounting rules for electrolytic hydrogen shape investor behavior and the resulting divergence between certified and actual emissions outcomes. The investor-centric, nested optimization framework is a valuable complement to system-planning studies, because it explicitly captures private incentives and strategic responses to accounting constraints.

That said, several remaining assumptions substantially affect the credibility and generality of the quantitative results and, in particular, the policy interpretation. I therefore recommend major revision.

Major comments (major revision)

1. Extrapolation from historical hourly data

The analysis extrapolates long-run outcomes from historical hourly price and emissions patterns. In high-renewables futures, hourly dynamics are likely to differ qualitatively (e.g., more frequent

negative prices, higher volatility, altered price–emissions correlations), which could materially change optimal operation and emissions outcomes.
The authors should either demonstrate robustness using at least one forward-looking or stylized hourly scenario that alters the joint distribution of prices and emissions, or explicitly limit quantitative conclusions to the historical-hourly setting.

As suggested, we have extended our analysis to include a forward-looking scenario for prices and hourly emission rates of general grid electricity using the Cambium dataset from NREL (2024, 2025). Cambium combines a capacity-expansion model (ReEDS) with an hourly production-cost model (PLEXOS) to project future grid operations, including hourly cost and emission metrics under alternative transition pathways. The projections include changes in the hourly generation mix and the associated average emission rates. Hourly electricity wholesale prices can be approximated by the projected short-run marginal system costs (i.e., the parameter *total_cost_busbar*).

Our calculations, detailed in Supplementary Note 8, show that the life-cycle performance of PtG systems based on Cambium is close to that based on our extrapolated scenarios for most variations considered. In particular, the results for the profitability of PtG Systems and the life-cycle average carbon intensity of hydrogen based on Cambium form a close lower envelope to those based on our scenarios. This partly reflects that the Cambium scenario projects a deeper decarbonization of the grid by 2035 than our extrapolation, leading to systematically lower future emission intensities. This also reflects that the projected marginal system costs in Cambium decline on average while their hourly variability increases, providing incentives for flexible operation without materially altering profitability outcomes.

2. Emissions attribution: average versus marginal effects
Key conclusions about "actual" emissions hinge on the choice of emissions factor. When additional electricity demand affects dispatch, average emissions may diverge substantially from marginal impacts.
The manuscript would benefit from clearer justification of the chosen accounting approach and from a sensitivity analysis using marginal emissions (or a defensible proxy), with discussion of implications for the certified–actual divergence..

We agree that the carbon intensity of hydrogen produced depends on the emission factors for grid electricity. Our main specification uses average emission factors because regulatory guidelines, including the U.S. Inflation Reduction Act, generally rely on these factors. To clarify this in the main body of the paper, we have added a sentence to the penultimate paragraph in Section 2.

To explore the impact of marginal emission factors for grid electricity, we repeated our analysis for the main specification using the Cambium dataset from NREL (2024). As detailed in Supplementary Note 13, our calculations show that the profitability of PtG systems and the policy impact of the tax credit based on either marginal or average emission rates are almost identical. In contrast, the life-

cycle average carbon intensity of hydrogen is significantly higher when marginal rather than average emission rates are used, simply because marginal emission rates are at least as high as average emission rates. We further find that the relative impact of different carbon accounting rules remains unchanged across all variations considered.

3. Exogenous grid assumption

Electricity prices, dispatch, and emissions are treated as exogenous. At scale, PtG deployment may affect all three, which is central to assessing policy effectiveness and actual emissions outcomes.

Either a simplified endogenous grid-response representation should be introduced, or the policy conclusions should be more tightly bounded to settings in which grid feedbacks are negligible.

In response to this point, we have reviewed the manuscript to ensure that our policy conclusions are properly stated in the context of the scenarios examined.

We also note that, because of the potential endogeneity effects, we state in the Conclusion that it would be instructive for future research to develop a (partial) market equilibrium model that maximizes total welfare for given price elasticities of electricity and hydrogen demand. Such a model would enable the analysis of how alternative carbon accounting rules for assessing the carbon intensity of hydrogen drive the total emissions resulting from electricity and hydrogen production, as well as the total tax credit payouts and their impact on a government's budget.

While our analysis takes the perspective of a representative investor seeking to maximize the net present value of investments in PtG systems, such an analysis has been missing in the literature, as pointed out in a recent review article (Brattle Group, 2024).

4. Cross-regional generalizability

Claims about generalizability across states depend critically on renewable resource profiles and their covariance with prices and emissions. Varying prices and emissions while holding capacity factors fixed risks biasing profitability and emissions comparisons.

This limitation should be addressed using state-specific capacity-factor time series where possible, or more clearly acknowledged in the interpretation of cross-state results.

As we state in Supplementary Note 9, our calculations for other geographic regions already substitute the respective hourly vectors for local (i) electricity selling and buying prices, (ii) capacity factors of wind and solar energy, and (iii) carbon intensity levels of general grid electricity. The differences in these vectors materially affect the life-cycle performance of PtG systems, as discussed in detail in the Supplementary Note.

5. Credit-forgoing behavior at high hydrogen prices
The manuscript highlights that investors may rationally forgo tax credits and rely on grid electricity at high hydrogen prices. This mechanism is potentially important for policy design but is currently described only qualitatively.
Quantifying the conditions under which this behavior emerges would strengthen the argument.

We note that Section 3 already discusses this effect quantitatively in connection with equations (3) and (6). To further illustrate the hourly operation of PtG systems, we have added Supplementary Figures 1-3 in Supplementary Note 2. These figures show the hourly operation of PtG systems for a selection of hours at hydrogen prices of $1.5/kg, $2.5/kg, and $3.5/kg. In particular, these figures show that, under carbon accounting rule A, PtG systems are incentivized to forgo the tax credit in some hours in favor of a higher conversion of general grid power. This effect occurs more frequently at higher hydrogen prices.

6. Operational realism: cycling and degradation
Different accounting rules imply different utilization and cycling patterns, which may affect electrolyzer degradation and replacement. Ignoring these effects could bias IRR comparisons across rules.
At least one sensitivity case incorporating simplified degradation or cycling costs would help assess the robustness of the main conclusions.

We agree that alternative carbon accounting rules may affect the usage-based degradation of PtG systems differently. Since our techno-economic model relies on a time-based degradation of PtG systems of 1.0% per year, we estimate the magnitude of potential differences in usage-based degradation between carbon accounting rules by repeating our main analysis for annual degradation rates of 0.5% and 2.0%. As detailed in Supplementary Note 10, we find that the profitability of PtG systems is only slightly higher (lower) when the annual degradation rate is lower (higher), reflecting that the operational capacity becomes slightly more (less) productive. The life-cycle average carbon intensity of hydrogen and the policy impact of the tax credit remain almost unchanged.

7. Interpretation of policy metrics
The "Policy Impact" metric measures hydrogen output per unit subsidy and may appear favorable under relaxed rules even when emissions are high. This risks being misinterpreted as an environmental performance indicator.
The manuscript should clarify the interpretation and limits of this metric and, ideally, complement it with an emissions-normalized measure (e.g., subsidy per unit of emissions abated).

We note that, contrary to the reviewer's interpretation, the policy impact of the tax credit is generally higher under the more stringent carbon accounting rules based on hourly electricity matching (see Figure 6). These rules generally lead to lower life-cycle average carbon intensity levels

for hydrogen than the less stringent rules based on annual electricity matching (see Figure 5). As such, we believe that our metric should not be prone to the risk of misinterpretation mentioned by the reviewer.

8.Minor comments
Please double-check consistency of variable names, units, and definitions across the main text and Supplementary.
Where time-sensitive regulatory timelines are mentioned, please ensure citations are provided and wording is appropriately bounded.

We have carefully reviewed the manuscript and Supplementary Information to ensure consistency of variable names, units, and definitions. In doing so, we have paid particular attention to citations and wording regarding regulatory timelines.

In conclusion, we again thank the two reviewers for their many insightful comments and suggestions. We believe that the comprehensive revision has resulted in a more comprehensive and transparent manuscript with sharper and more robust findings. We hope you agree.

Response to Reviewer 3

The overall structure of the manuscript is clear, the methodology is rigorous, and the core conclusions are consistent with the modeling framework. I recommend a minor revision to further improve the quality of the paper :
Figure 1 is helpful conceptually, but its visual design could be improved for better clarity and aesthetic quality.

As suggested by Reviewer #3, we revised Figure 1 to enhance its clarity and visual appeal. Specifically, we refined the layout, incorporated illustrative icons, and added annotative labels together with a legend. We are confident that this revision improves the overall presentation of the manuscript.
